# The Design of Intrinsically Conductive Metal‐Organic Frameworks for Thermoelectric Materials

**DOI:** 10.1002/smsc.202400469

**Published:** 2025-01-27

**Authors:** Molly McVea, Christian B. Nielsen, Oliver Fenwick, Petra Ágota Szilágyi

**Affiliations:** ^1^ School of Engineering and Material Science Queen Mary University of London London E1 4NS UK; ^2^ Department of Chemistry Queen Mary University of London London E1 4NS UK; ^3^ Centre for Materials Science and Nanotechnology (SMN) Department of Chemistry University of Oslo P.O. Box 1033 Blindern N‐0315 Oslo Norway

**Keywords:** electrical conductivity, intrinsically conducting porous materials, metal‐organic frameworks, seebeck coefficient, thermal conductivity, thermoelectricity

## Abstract

Thermoelectric (TE) materials offer exciting promise in the development of sustainable, green energy alternatives. Ideal TE materials promote high electrical conductivity, whilst effectively scattering phonons for reduced thermal conductivity, thus giving the phonon‐glass electron‐crystal concept. Metal‐organic frameworks (MOFs) have emerged as a versatile class of materials that could meet these criteria. The high crystallinity of MOFs can offer effective pathways for charge transport whilst their intrinsic high porosity yields ultralow thermal conductivity. The high structural diversity of MOFs, owing to the versatile coordination of metal cation/cluster and linker offers the potential to systematically tune their properties for TE performance. This review examines the advancement in the design strategies that have thus far been implemented toward intrinsically conductive MOFs and how these should be tailored for application toward TE materials. By addressing the challenges and leveraging the unique properties of MOFs, future research can pave the way for innovative and efficient TE MOF materials.

## Graphical abstract

Owing to their high crystallinity combined with high porosity, metal‐organic frameworks (MOFs) appear as a promising class of materials for thermoelectric applications, potentially offering the ideal combination of high electrical and low thermal conductivity. This review explores intrinsically conducting MOFs and design strategies for enhancing their thermoelectric performance metrics.

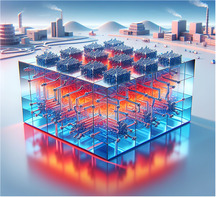

## 1 Introduction

June 2023 reported the warmest ever recorded global temperature average and, as a whole, 2023 was the hottest year in global temperatures ever recorded: 1.48 °C warmer than pre‐industrial times.^[^ ^1^, ^2^, ^3^ ^]^ This is a direct result of the crippling dependence humans have had on unsustainable fossil fuels. This anthropogenic climate change has already led to severe impacts globally, including extreme weather conditions. This comes as leading experts warn that to achieve the 1.5 °C global warming limit agreed upon within the framework of the Paris Agreement, greenhouse gas emissions must peak by 2025.^[^ ^3^, ^4^ ^]^ Despite this, the global energy demand continues to rise along with the consumption of non‐renewable fossil energy carriers (coal, gas, and oil). However, recent estimates indicate that 2023 could mark the turning point for decreased fossil fuel dependence, as the share of electricity generated from renewable sources has grown to almost 40% within the EU and up to 30% globally.^[^ ^5^, ^6^ ^]^ This is a result of a significant increase in wind and solar power production, as well as a drive toward accelerated electrification.^[^ ^7^ ^]^ Nevertheless, electricity generation is still the most significant contributor to global CO_2_ emissions, so the need to develop the means to generate electricity sustainably and efficiently is paramount.^[^ ^8^ ^]^

Maximizing the efficiency of electrical power sources is critical to tackling the growing global energy demands. Estimates indicate that as much as 50% of industrial energy input is lost as heat. Given that the industrial sector accounted for 37% of total global energy consumption in 2022, upwards of 18.5% of the total global energy demand is lost as heat in the industrial sector alone.^[^ ^6^, ^9^, ^10^, ^11^ ^]^ Developing strategies to convert this waste heat to useful electricity will vastly improve energy efficiency, thus aiding the growing energy demand without the need for more non‐renewable sources.^[^ ^12^ ^]^

Thermoelectric (TE) devices can generate clean electrical energy via the conversion of heat in the form of temperature gradients. As such, TE devices offer a promising solution for harvesting waste heat energy to achieve more efficient and sustainable energy generation.^[^ ^12^ ^]^ This, in turn, could lessen dependence on fossil fuels by providing an alternative clean energy approach with great energy efficiency, which would curtail CO_2_ emissions, provided a high enough conversion can be achieved.^[^ ^13^ ^]^

The maximum theoretical efficiency of waste heat conversion of TE generators is determined by the dimensionless figure of merit *zT* of the thermoelectric materials they contain (Equation (1)).

(1) $$zT=\;\frac{S^{2}\sigma T}{\kappa }$$

where *S* is the Seebeck coefficient, *σ* is the electrical conductivity, *T* is the absolute temperature, and *κ* is the thermal conductivity. When developing TE materials, the principal aim is to maximize the *zT* value. While there is no known theoretical limit to *zT*, the highest *zT* values for state‐of‐the‐art commercial TE materials are around *zT* = 1.^[^ ^12^ ^]^ The challenge for maximizing the *zT* value arises from the inter‐dependence of the aforementioned thermoelectric parameters: *S*, *κ*, and *σ*. High *zT* values will arise from materials with low thermal conductivities and high electrical conductivities. This gives rise to the ideal concept of “phonon‐glass electron‐crystal” materials. This describes a material that allows for the efficient transport of electrons (charger carriers) while effectively scattering phonons.

Current TE devices rely on materials of rare and environmentally harmful elements (e.g., Bi, Te, Pb).^[^ ^14^ ^]^ Their inorganic nature also limits the synthesis and processibility, meaning that despite some materials having yielded *zT* values > 2, they remain too expensive or impractical for many wider applications.^[^ ^12^, ^14^, ^15^, ^16^, ^17^ ^]^ In recent years, this has led to research focused on designing alternative TE materials, such as conductive organic polymers.^[^ ^18^ ^]^ Whilst organic materials allow for ease of processing and offer the potential to tune thermal and electrical properties, they tend to lack the long‐range order seen in inorganic TE materials. As a result, organic materials typically have poor charge‐carrier mobilities, consequently only affording low thermoelectric power factors ($S^{2}\sigma )$. They are, however, typically regarded as more sustainable and less toxic than inorganic materials, whilst also offering lower material cost and mechanical flexibility.^[^ ^15^, ^19^ ^]^ Consequently, they are deemed appealing for developing devices to tackle the ongoing environmental crisis, as well as other wearable device applications.^[^ ^19^ ^]^ Nevertheless, both inorganic and organic‐based TE materials are still limited by poor efficiency and require substantial optimization to enable widespread integration in commercially viable devices.^[^ ^15^, ^20^, ^21^, ^22^, ^23^ ^]^

Metal‐organic frameworks (MOFs) are highly porous, hybrid organic‐inorganic crystalline materials formed by the self‐assembly of inorganic nodes connected by organic linkers via coordination bonds. It is suggested that MOFs’ intrinsically hybrid nature may render them an ideal candidate for synergistically combining the advantages of both classes of materials. The long‐range crystallinity can offer pathways for high electrical conductivity, while the organic nature of the linkers endows MOFs with high tunability, giving a wide range of structural diversity, as well as the opportunity to use abundant and low‐toxicity elements. The high porosity, range of atomic masses, and different bonding types of MOFs yield materials with ultralow thermal conductivities. The possibility of achieving intrinsically high electrical conductivity with intrinsically low thermal conductivity makes MOFs of particular interest for TE materials.

In many ways, MOF‐based TE materials bear many similarities to organic thermoelectric materials (OTE), a field that has garnered considerable research interest and seen significant advancements in recent years. OTE is a large and growing field with many extensive reviews, and as such full comparison with MOF TE is beyond the full scope of this review. However, it can be noted that MOF TE and OTE both rely heavily on the conjugation of organic molecules to promote electrical conductivity. Current OTEs are limited by modest Seebeck coefficient and charge mobility, both a result of structural and energetic disorder, for which MOFs could hope to address by the intrinsic incorporation of metal ions for manipulation of electronic structure and improved long‐order crystallinity.

## 2 Metal‐Organic Frameworks as Thermoelectric Materials

Traditionally, MOFs with 3D porous structures have been viewed as electrical insulators, with this inherent lack of electrical conductivity inhibiting their development in electronic devices.^[^ ^24^, ^25^, ^26^, ^27^ ^]^ However, the recent development of a series of 2D planar MOFs, often referred to as “metal‐organic graphene analogs (MOGs)”, which have demonstrated good electrical conductivities, has led to the emergence of research into their application in electronic devices and as electrocatalysts.^[^ ^28^, ^29^, ^30^, ^31^, ^32^, ^33^, ^34^, ^35^, ^36^, ^37^ ^]^

Whilst these 2D MOFs exhibit low thermal yet good electrical conductivities, their TE performances (*zT*) thus far reported are still considerably lower compared to incumbent inorganic TE materials.^[^ ^38^, ^39^, ^40^, ^41^ ^]^ This has been chiefly a result of the low values of the Seebeck coefficient, most likely hindered by a lack of theoretical understanding, consequently limiting engineering of the band structures and electrical transport mechanisms in MOFs.^[^ ^38^, ^39^, ^40^, ^41^ ^]^

For MOFs to become viable as TE materials, more work must be done to develop a better understanding of their structure–function relationships. Understanding the origin of the electronic band structure and charge‐carrier transport pathways will help with the systematic design of new linkers, optimized metal selection, and pre‐ and post‐synthetic modifications. Finding optimized strategies for engineering MOFs tailored toward improved TE properties can pave the way for the crucial development of efficient, sustainable TE materials. Therefore, the motivation for this review is to explore the aforementioned properties of previously published MOFs to generate insight into the origins of intrinsic conductivity within MOFs and to spur further research into their application as efficient TE materials.

Section 2 presents our current understanding of how the thermoelectric parameters (*σ*, *S*, and *κ*) arise and can be manipulated within MOFs.

### 2.1 Thermal Conductivity of Porous Materials

The thermal conductivity of a material is defined as the material's ability to conduct heat and is dependent on the heat capacity (*C*), the velocity (*ν*_sound_), and the phonon mean free path (*l*), given by the relationship defined in Equation (2).

(2) $$\kappa =\;\frac{1}{3}C\nu_{\text{sound}}l$$

The total thermal conductivity (*κ*) for a given material arises from two contributions: the electronic thermal conductivity (*κ*_e_) and the lattice thermal conductivity (*κ*_l_).^[^ ^38^, ^42^ ^]^

(3) $$\kappa =\;\kappa_{e}+\kappa_{l}$$

The electronic component of the thermal conductivity is a consequence of heat transport by electrons and is directly proportional to the electrical conductivity according to the Wiedemann–Franz law (Equation (3)), where *L* is the Lorenz number.^[^ ^38^, ^42^, ^43^ ^]^

(4) $$\kappa_{e}=L\;\sigma \;T$$

As such, increasing the electrical conductivity, which is desirable to achieve high *zT* TE materials, will also lead to an increase in *κ*_e_, highlighting the interdependent nature of these parameters. The lattice component of the thermal conductivity, however, arises from the phonon contribution, and it is, therefore, *κ*_l_ minimization that is the focus for maximizing *zT*.

Typically, low *κ*_l_ has been engineered in TE materials via defects and grain boundaries.^[^ ^44^ ^]^ The presence of which enable phonon scattering for reduced thermal conductivity, however, this will often result in disruptions to the electronic pathways too, and thus lower the electronic conductivity as well.

In spite of the importance of *κ* in applications of MOFs, experimental measurements have been limited due to the challenges of measuring small crystal sizes. Additionally, the difficulties associated with the synthesis of large enough crystal samples have also hindered thermal conductivity measurements on single‐crystal MOFs. However, there is on‐going research in improving both MOF single‐crystal synthesis and techniques for more accurate thermal conductivity measurements on MOF polycrystalline samples.^[^ ^45^, ^46^ ^]^ It should thus be noted that conductivities reported in **Table** **1** were measured on polycrystalline samples (either pressed pellet powder or thin films) and thus are subject to grain boundaries and contact resistances between domains limiting the thermal conductivities.^[^ ^38^, ^40^, ^41^, ^45^, ^47^, ^48^ ^]^

**Table 1:** Summary of metal‐organic frameworks that have exhibited intrinsic electrical conductivity and thermoelectric behavior at room temperature (298–300 K).

MOF	Metal	Ligand	Interlayer Separation [Å]	Metal Oxidation States	Form	Measurement Methoda)	Conditions	*σ* [S cm^−1^]	S [μV K^−1^]	*κ* [W m^−1^ K^−1^]	PF [μW cm^−1^ K^−2^]	*zT*	Specific Surface Area [m^2^g^−1^]	Note	References
Ni_3_(HITP)_2_	Ni	HITP	3.3	–	PP	Van der Pauw	Vacuum	58.8	−11.9	0.21	8.3 × 10^−3^	0.0012	766	–	[38]
					TF			–	−50	–	–	–	–	–	
Ni_3_(HITP)_2_	Ni	HITP	<4.1	Ni (II)	PP	Two‐point probe	–	0.195	−13.4	–	3.5 × 10^−5^	–	251	–	[70]
Pt_3_(HITP)_2_	Pt		–	Pt (II)	PP			0.327	37.7	–	4.7 × 10^−5^	–	67	amorphous	
Pd_3_(HITP)_2_	Pd		–	Pd (II)	PP			0.261	19.6	–	1.0 × 10^−4^	–	148	amorphous	
Cu_3_(HHTP)_2_	Cu	HHTP	3.13	Cu (II)	TF	Four‐point Probe	Vacuum	2.28 × 10^−3^	−121.4	–	3.15 × 10^−5^	–	–	–	[71]
					PP			3.8 × 10^−3^	−7.24	–	2 × 10^−7^	–	–	–	
Ni_3_(PTC)	Ni	PTC	3.4	Ni (II)/Ni (III)	PP	Four‐point Probe	–	9	47	0.2	0.02	0.003	–	–	[40]
Cu_3_(HTB)_2_	Cu	HTB	3.4	Cu (I)	TF	Four‐point Probe	–	2000	−21	1.99	0.88	0.013	–	–	[41]
Cu_3_(HTB)_2_	Cu	HTB	3.38	Cu (I)/Cu	TF	Four‐point Probe	–	1580	−10	–	0.158	–	–	–	[47]
Ni_3_(HIB)_2_	Ni	HIB	3.2	Ni (II)	PP	Van der Pauw	Ambient	1	61.8	–	–	–	–	–	[72]
							Nitrogen	1	−65.1	–	–	–	–	–	
Co_3_(HIB)_2_	Co	HIB	3.2	Co (II)	PP		Ambient	0.2	<<10	–	–	–	–	–	
							Nitrogen	5	<10	–	–	–	–	–	
Cu_3_(HIB)_2_	Cu	HIB	3.2	Cu (II)	PP		Ambient	0.001	−340	–	–	–	–	–	
							Nitrogen	0.001	−240	–	–	–	–	–	
Zn_3_(HIB)_2_	Zn	HIB	–	Zn (II)	PP	Van der Pauw	Vacuum	0.86× 10^−3^	200	–	3.44 × 10^−5^	–	145	–	[48]
Ni_3_(HATI_C1)_2_	Ni	HATI_C1	6.78	Ni (II)	PP	Van der Pauw	Air	0.011	76.2	–	0.0064	–	480	–	[92]
Ni_3_(HATI_C3)_2_	Ni	HATI_C3	7.32					0.0045	387.2	–	0.0068	–	413	–	
Ni_3_(HATI_C4)_2_	Ni	HATI_C4	7.46					0.0009	423.8	–	0.00162	–	385	–	

a) The electrical conductivity measurement method could influence relative results for comparison, as discussed in the review by Xie et al.^[^ ^57^ ^]^

MOFs typically have reported low thermal conductivities (<2 W m^−1^ K^−1^) without the need for any defect engineering.^[^ ^43^ ^]^ These intrinsically low thermal conductivities can be attributed to a variety of structural factors including high porosity, low structural density, complex unit cells, bond heterogeneity, and large mass mismatching. These all result in enhanced phonon scattering, contributing to intrinsically low κ_l_ values.

#### 2.1.1 Effect of Porosity and MOF Density on Thermal Conductivity

**Figure 1:**
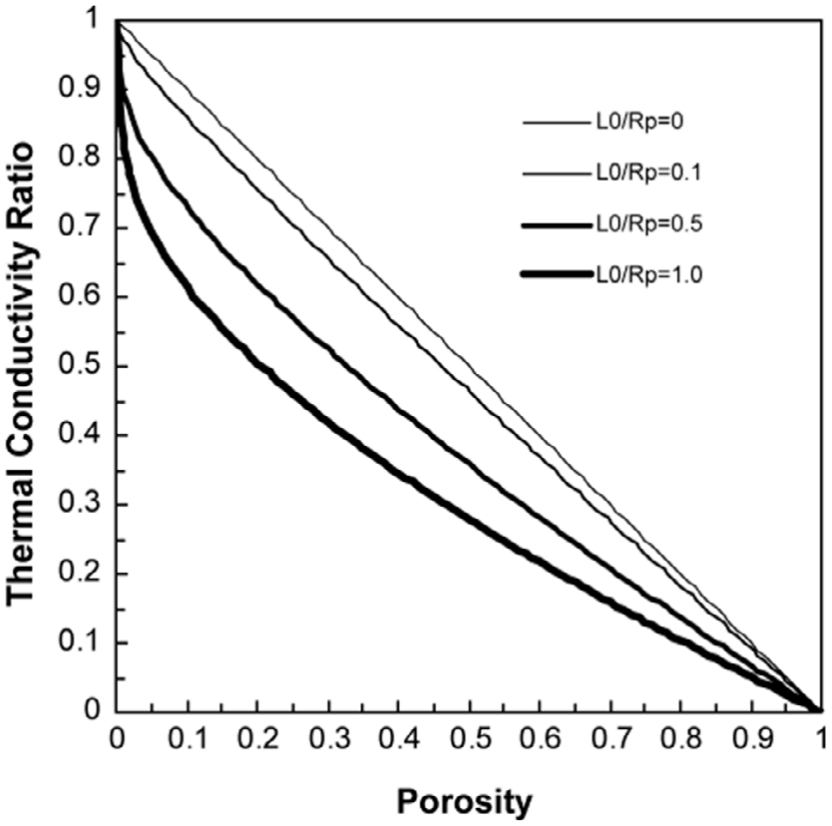
Ratio of the thermal conductivity of a porous sample (*κ*) to its non‐porous counterpart (*κ*_0_) variation with porosity, for a series of systems with increasing phonon mean free path (*l*_*0*_) to pore size (*R*_p_). Reprinted with permission.^[^ ^49^ ^]^ Copyright 2006, Springer Nature.

The intrinsic high porosity of MOFs yields materials with high surface areas, low densities, and, consequently, low thermal conductivities. This is due to the enhanced phonon scattering at the pore boundaries (gas–phonon interactions and boundary scattering) reducing the phonon mean free path (*l*). Hence, the higher the porosity, and larger the surface area, the lower the thermal conductivity is expected to be. Despite this, there is a lack of reports detailing the dependence of a material's thermal conductivity on its porosity. A simplified model, Equation (5), developed by Sumirat et al. via the manipulation of Equation (2), demonstrated the significant impact the material's porosity (*ф*), defined as the ratio of the total volume of the pores to the material volume, has on tailoring the thermal conductivity, **Figure** **1**.^[^ ^49^ ^]^ It also highlights the importance of the pore size (*R*_p_) relative to the mean free path of phonons in the nonporous material, *l*_0_. *κ*/*κ*_0_ is the ratio of the thermal conductivity to the thermal conductivity of the nonporous material, and increasing porosity significantly contributed to the reducing *κ*/*κ*_0_, Figure 1, with this effect increasing with the *l*_0_/*R*_p_ ratio. From this, it was determined that in order to yield materials of significantly low thermal conductivities, pore sizes that are in a similar magnitude to that of the phonon mean free path (*l*_0_/*R*_p_ = 1) are ideal, as well as high porosity.^[^ ^49^ ^]^ MOFs are associated with short phonon mean free paths, typically only a few nanometers, which is similar in magnitude to the typical MOF pore sizes (*l*_0_/*R*_p_ = 1), contributing to the ultralow thermal conductivity observed in these materials.^[^ ^50^ ^]^

(5) $$\frac{\kappa }{\kappa_{0}}=\frac{1-\varphi }{1+\frac{l_{0}}{R_{p}}\varphi^{\frac{1}{3}}}\;$$

**Figure 2:**
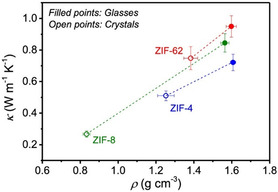
Correlation between framework density (*ρ*) and thermal conductivity (*κ*) as determined from MD simulations for the glass (filled points) and crystal (open points) analogues of ZIF‐4 (blue), ZIF‐8 (green), and ZIF‐62 (red). Reprinted (adapted) with permission.^[^ ^46^ ^]^ Copyright 2020, American Chemical Society.

**Figure 3:**
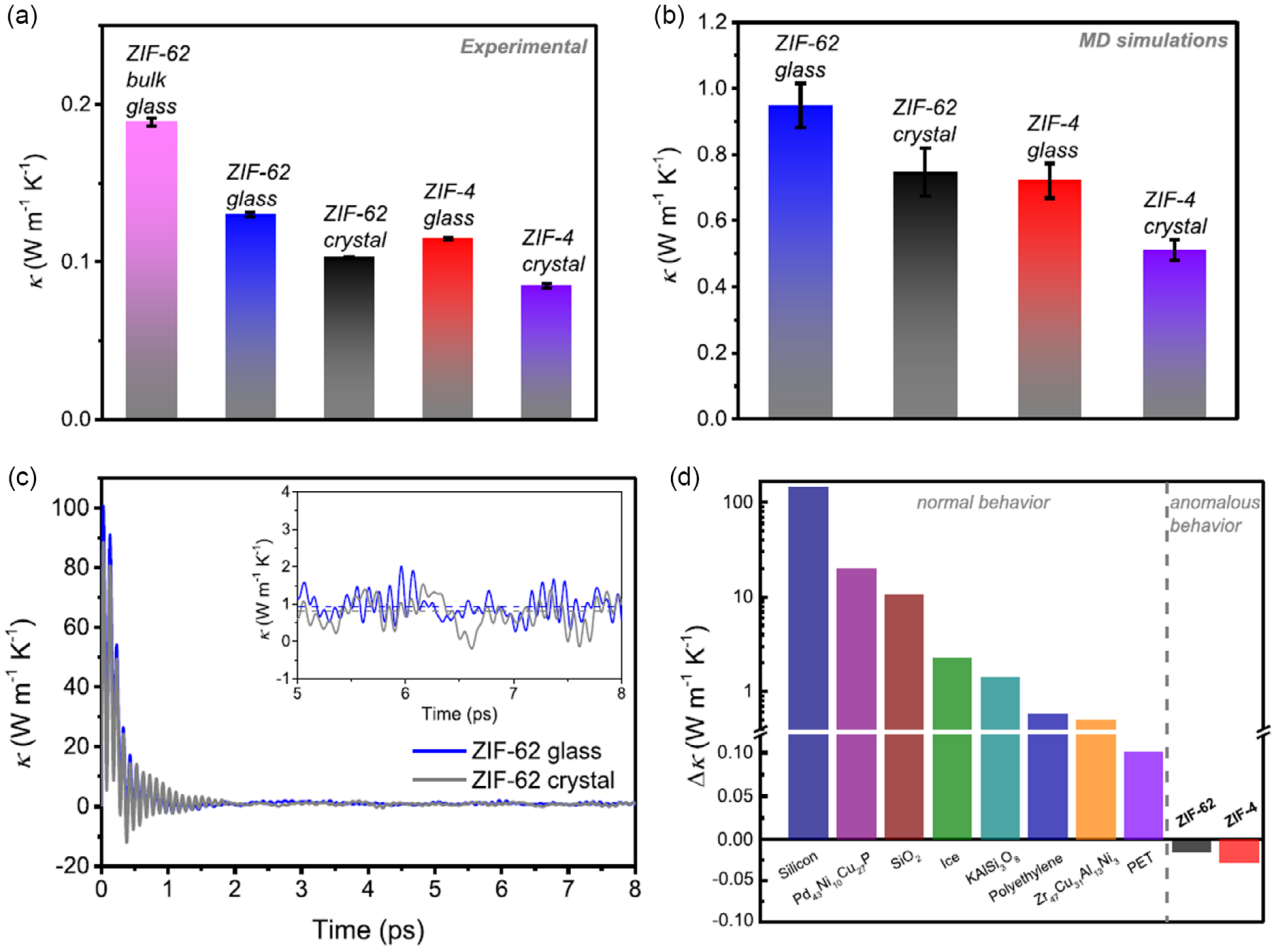
a) Experimental thermal conductivity measurements determined from powder pellets and ZIF‐62 bulk glass samples. b) Theoretical thermal conductivity values determined via molecular dynamics (MD)‐simulations. c) Cumulative *κ*‐values based on the running integral of the heat current autocorrelation function. The inset shows the used convergence region from 5 to 8 ps, where the dashed line represents the average *κ*‐values. d) The change of *κ* values (Δ*κ* = *κ*_crystal_ *– κ*_amorphous_) of 10 different materials between their amorphous and crystalline states—for comparison with ZIF results. For all materials, bar ZIFs, the thermal conductivity of the crystalline material was greater than that of the amorphous. Reprinted (adapted) with permission.^[^ ^46^ ^]^ Copyright 2020, American Chemical Society.

The effectiveness of the intrinsic high porosity and low structural density of MOFs in suppressing thermal conductivity has been demonstrated by comparison of a series of single‐crystal zeolitic imidazolate framework (ZIF) MOFs to their ZIF glass analogs.^[^ ^46^ ^]^ ZIF glasses, upon melting, lose the nano‐porosity typically associated with crystalline ZIFs. As a result, these amorphous ZIF glasses all yielded higher densities and higher thermal conductivities with respect to the single‐crystal equivalents, **Figure** **2**. This is contrary to what is typically expected of crystalline polymorphs, highlighting the unusually low thermal conductivity of the crystalline ZIFs–a result of their low structural density and high porosity.^[^ ^46^, ^51^ ^]^ This phenomenon was demonstrated both experimentally, with powder pellets of the ZIF glasses and crystals (**Figure** 2 and **3**a), and computationally (Figure 3b,c). Figure 3d compares the typical change in thermal conductivity associated with amorphous and crystalline equivalents to these ZIF materials. Typically, amorphous samples are expected to produce lower thermal conductivities, as demonstrated in Figure 2, due to an increase in the disorder‐induced phonon‐phonon scattering. Whilst the ZIF glasses still demonstrate such disorder‐induced phonon scattering and, consequently, still yield exceptionally low thermal conductivities, their crystalline equivalents yield even lower due to the larger porosity.^[^ ^46^ ^]^

Therefore, it can be concluded that good crystallinity is significant in imparting the high porosity associated with MOFs, and thus yielding low thermal conductivity frameworks. As such, unlike traditional TE materials, good crystallinity could be favorable for minimizing *κ*_l_ for optimized *zT*.

#### 2.1.2 Effect of Pore Size and Shape on Thermal Conductivity

As touched upon earlier, the degree of porosity of a MOF is also dependent on the size and shape of the MOF pores, which each have their own impact on the overall thermal conductivity.

**Figure 4:**
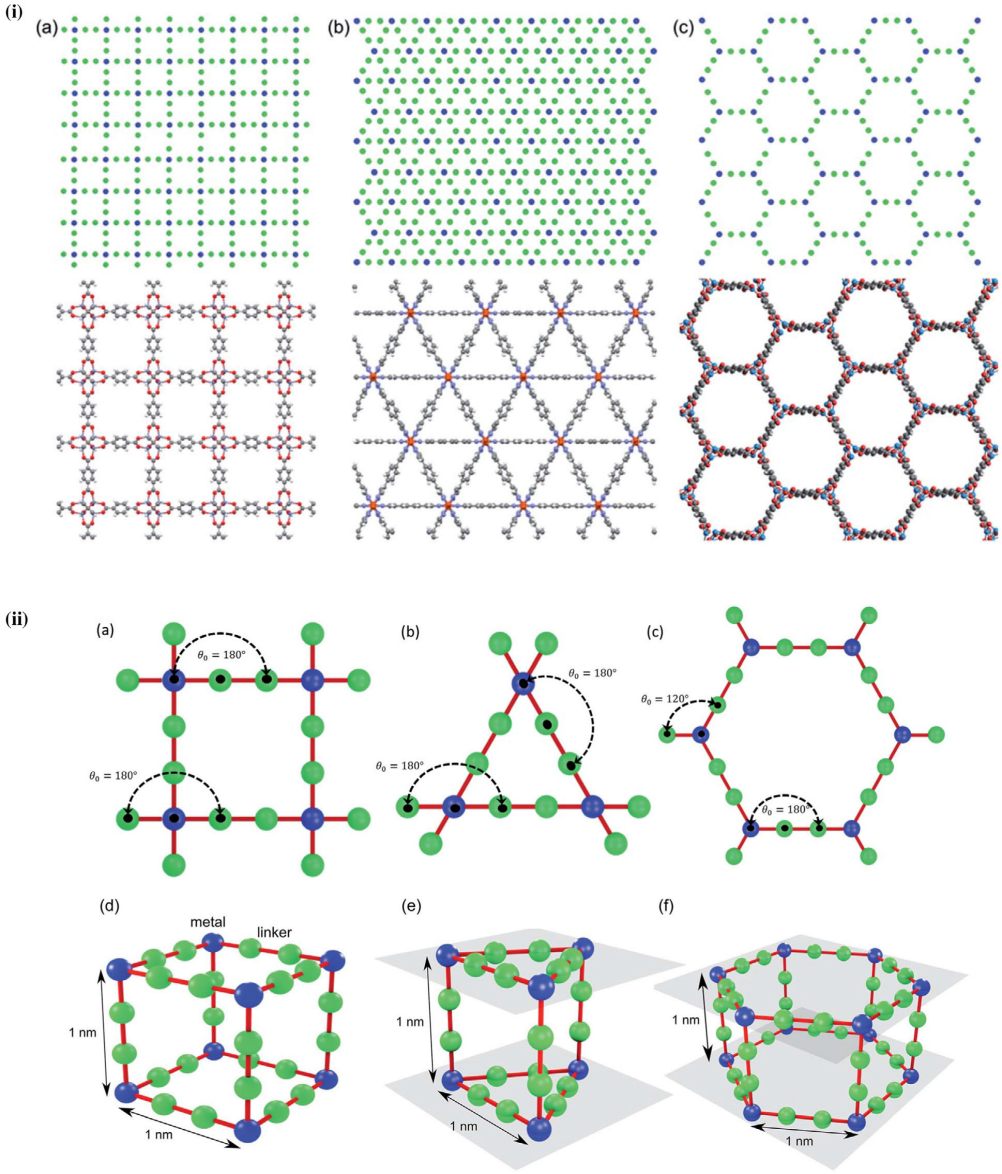
i) Cross‐sectional views of idealized MOFs for theoretical study with a) simple cubic pores—based on IRMOF‐1, b) triangular‐channel pores—based on Fe_2_(BDP)_3_, and c) hexagonal‐channel pores—based on MOF‐74. ii) Graphical description of parameters used for angles, bonds (top), and a,d) interplane spacing for the unit cells of simple cubic pores, b,e) triangular‐channel pores, and c,f) hexagonal‐channel pores used for theoretical study.^[^ ^52^ ^]^ Reproduced with permission.^[^ ^52^ ^]^ Copyright 2017, Royal Society of Chemistry.

**Figure 5:**
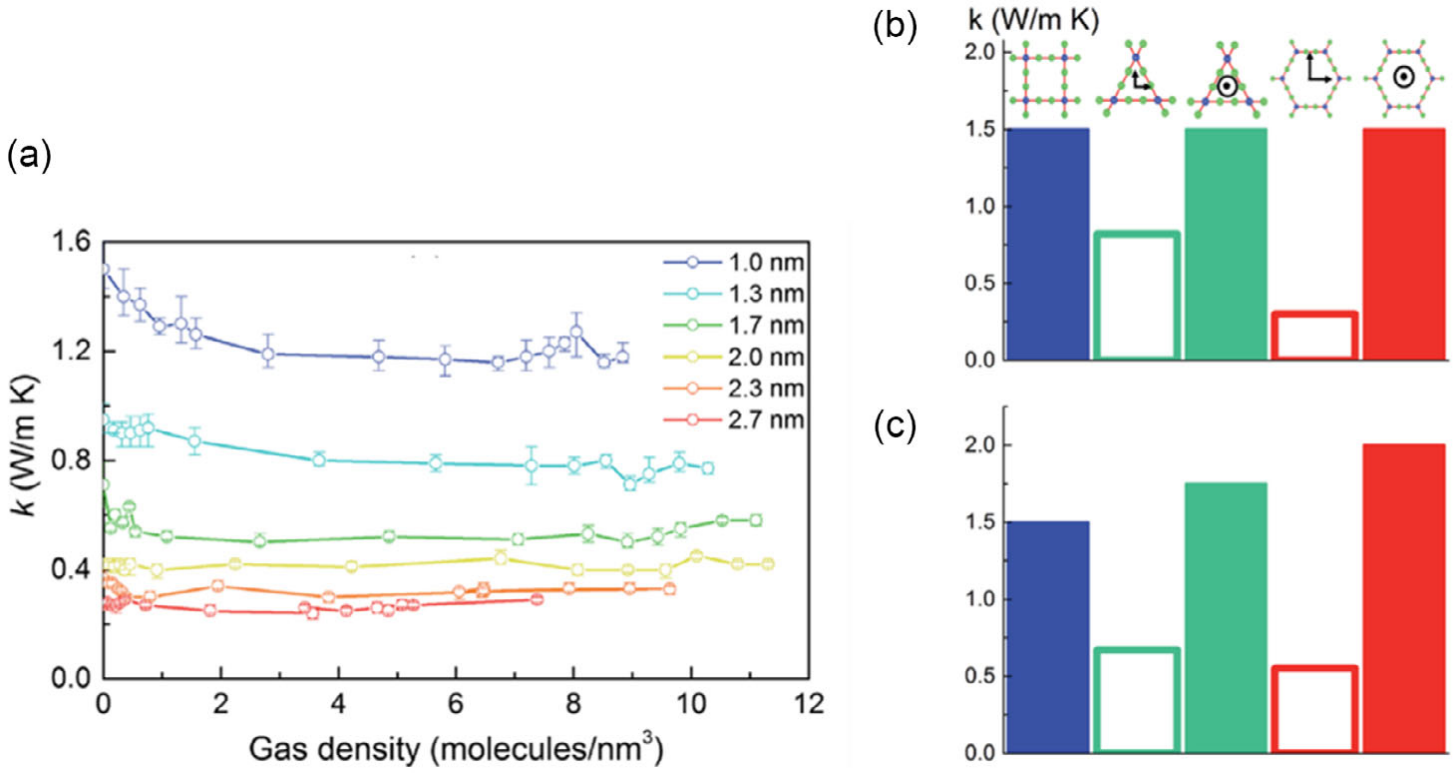
a) Variation of thermal conductivity with pore size for gas‐loaded simple cubic MOFs as a function of gas density. b,c) Calculated thermal conductivities for MOFs with different pore shapes (blue: simple cubic, green: triangular (open bar—perpendicular to channel, filled bar—parallel to channel), red: hexagonal (open bar—perpendicular to channel, filled bar—parallel to channel). For comparison, MOFs with the same (b) linker size (1 nm) and (c) cross‐sectional area (1 nm^2^).^[^ ^52^ ^]^ Reproduced with permission.^[^ ^52^ ^]^ Copyright 2017, Royal Society of Chemistry.

Moreover, the organic linkers of MOFs offer the potential to systemically tune the structure for the desired effect in specific properties, and this allows the ability for structural engineering toward viable thermal conductivities. Babaei et al. investigated the impact of tuning the pore size and shape via different linker sizes and bonding geometries in a computational study, **Figure** **4**.^[^ ^52^ ^]^ It was found that, as the size of the pore increases, the predicted thermal conductivity decreases (**Figure** **5**), attributed to the decreased areal density of bonded interactions. Babaei et al. also investigated the effect of pore shape, cubic, triangular, and hexagonal (Figure 4), on the thermal conductivity (Figure 5b,c).^[^ ^52^ ^]^ This showed that for the same linker size (1 nm), the thermal conductivity in the parallel direction (to the pore channel) is the same for all three structures (Figure 5b,c, filled bars). However, while cubic pores demonstrated no difference between the parallel (to direction of pore) and perpendicular (to direction of pore) thermal conductivities, there was significant anisotropy for triangular pores, and even more so for hexagonal pores (Figure 5b,c). Whilst hexagonal pores reported the highest parallel thermal conductivity for pores of the same cross‐sectional area (1 nm^2^), the perpendicular thermal conductivity was the lowest.^[^ ^52^ ^]^

In conclusion, careful selection of pore shape and size, as well as manipulation of the MOF orientation, could help minimize the effects of thermal conductivity. Larger pore sizes are favored for reducing thermal transport. When considering the most effective pore shape for reducing thermal conductivity, thought must also be given to the direction—with hexagonal and triangular pores demonstrating significant thermal conductivity anisotropy compared to cubic pores. Hexagonal pores, measured perpendicular to the pore channel, are most adept for reducing the thermal conductivity.

#### 2.1.3 Effect of Atmosphere on Thermal Conductivity

The high porosity typically associated with MOFs has deemed them favorable for hosting guest molecules within the pores, a practice heavily utilized in various other MOF applications. Whilst the effect of hosting electroactive guest molecules within the pores for the purpose of doping is beyond the scope of this review, consideration of atmospheric adsorbates on the TE properties is still of importance.

**Figure 6:**
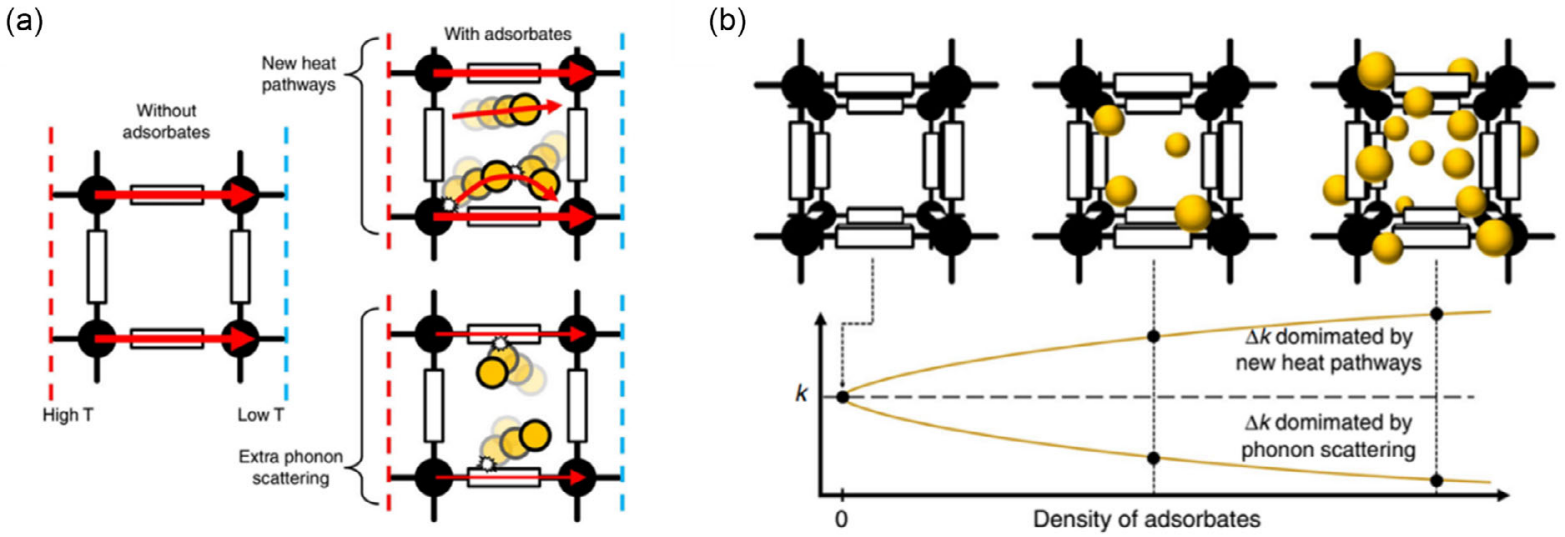
a) Graphical representation of the influence adsorbates can have on phonon transfer in MOFs. Without the presence of any adsorbates, thermal conductivity may only occur through the MOF framework; addition of adsorbates within the MOF pores can either result in new heat transfer pathways (increasing thermal conductivity) or increased phonon scattering via adsorbate‐framework collisions (decreasing thermal conductivity). b) As the density of adsorbates increases, the thermal conductivity can either increase or decrease depending on whether the effect of new phonon pathways or more phonon scattering (respectively) is more dominating.^[^ ^53^ ^]^ Reprinted with permission.^[^ ^53^ ^]^ Copyright 2020, Nature Communications.

The computational study by Erickson et al. suggested two opposing effects of adsorbates within MOF pores, **Figure** **6**. The presence of guest molecules could offer either a pathway for improved thermal conductivity or increased phonon scattering, depending on the MOF‐adsorbate interactions. Which of these is dominating in a given system should indicate whether there is an increase or decrease in the observed system's thermal conductivity and likely is dependent on the interaction between the MOF and given adsorbate.^[^ ^53^ ^]^

Two studies found the thermal conductivity to be dependent on the gas density within the pores. At low gas density, the thermal conductivity was lower due to the effect of weakly interacting guest molecules, which increased the frequency of phonon scattering via “rattler” modes. However, as the density of the guest is increased, thermal conductivity increases also up until a point where the gas itself becomes the main contributor to the thermal conductivity.^[^ ^52^, ^54^ ^]^ This suggests that strongly interacting guest molecules could increase the thermal conductivity by providing alternative heat transport pathways. Interestingly, MOFs with smaller pores (<1.7 nm) also demonstrated a greater dependency on the density of gas molecules within the pores, and the largest pores saw virtually no difference in thermal conductivity due to changes in the gas density.^[^ ^38^ ^]^

**Figure 7:**
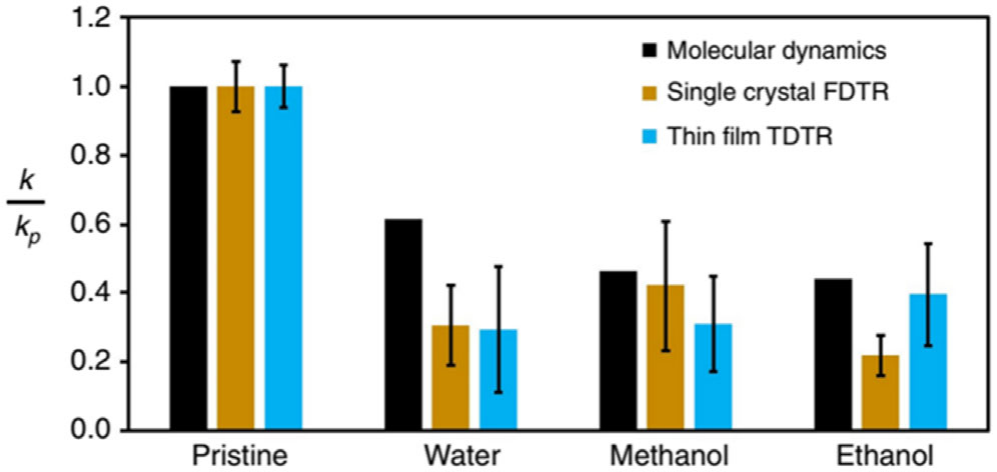
Normalized thermal conductivity measurements for pristine HKUST‐1 with water, methanol, and ethanol as adsorbates were measured as single crystals and thin films compared to computational models.^[^ ^53^ ^]^ Reprinted with permission.^[^ ^53^ ^]^ Copyright 2020, Nature Communications.

A separate study observed both experimentally and computationally, a decrease in thermal conductivity in the presence of adsorbates (water, methanol, and ethanol) in HKUST‐1 (**Figure** **7**).^[^ ^53^ ^]^ Not only did the incorporation of adsorbates within the MOF reduce the intrinsic thermal conductivity of the MOF, but the thermal conductivity of the bulk adsorbate was also reduced, which was attributed to the MOF geometric confinement.

The effect of different atmospheres on the MOF's thermal conductivity could thus be dependent on the interaction of the adsorbate with the MOF surface, thus warrants further careful evaluation in order to be used as a system design principle for a particular TE application.^[^ ^53^ ^]^

#### 2.1.4 Effect of Metal Node and Linker Acoustic Mismatch on Thermal Conductivity

MOFs also afford ultralow thermal conductivities due to their chemical complexity. The presence of a ‘thermal bottleneck’ at the interface between the organic linker and inorganic node has been attributed to an acoustic mismatch arising from a mass difference between the node and linker atoms.^[^ ^55^ ^]^ Two modes of heat transport were identified in MOF‐5 (and are applicable to all MOFs): heat transfer between localized modes and propagation through lattice modes. It was found that the linker exhibits the lowest thermal resistance, while the inorganic nodes show equal or greater resistance. However, the greatest hinderance to heat flow occurs at the interface between the two, creating a “thermal bottleneck”.^[^ ^55^ ^]^ However, this study only accounted for metal–oxygen bonds of non‐conductive frameworks, which might not fully account for the metal–linker interactions for relevant in TE MOFs.

The acoustic mismatch of the linker and nodes was systematically evaluated by varying the node masses. It was found that nodes with heavier masses, and hence a higher mass mismatch with the linker atoms, increased the thermal resistance at the linker‐node interface and had lower thermal conductivities. On the contrary, changing the linker impacted primarily the molecular modes, having negligible effect on the lattice modes and thus a smaller change in the thermal conductivity.^[^ ^55^ ^]^

In addition, the acoustic mismatch highlighted the importance of the nature of the linker–node coordination bond via comparison of Mg, Ca, and Zn nodes. While increasing mass (Mg to Ca, Mg to Zn) correlated with an expected decrease in the thermal conductivity, due to an increase in the mass mismatch with ligand, this was not the case for Ca to Zn. The significantly lower thermal conductivity for the MOF with the Ca node was attributed to the much weaker Ca—O bond (2.25 Å) compared to Mg (1.96 Å) and Zn (1.95 Å).^[^ ^55^ ^]^

As such, design strategies for optimization toward the desired thermal conductivity of MOFs could involve selectively tuning the node–linker bond length and strength, and maximizing the node‐to‐linker mass difference. For achieving low thermal conductivities, this will tend toward weaker, longer bonds with larger, heavier metal nodes; however, potential negative impacts on the electrical conductivity would also have to be considered for TE applications.

Despite the high‐level interest in MOF thermal conductivities, especially for application in adsorptive gas storage and catalytic and thermoelectric applications—there is still a limited understanding of the structure–property relationship in MOF thermal transport. Whilst the aforementioned studies indicate the significance that porosity, density, pore size and shape, absorbates, and metal node–linker bonding interactions affect the thermal conductivity, they are limited by their relatively small study size. However, Islamov et al. recently completed a high‐throughput computational study of over 10 000 hypothetical MOF crystals, seeking to gain a fundamental understanding of the structure–property relationship of MOFs and thermal transport.^[^ ^56^ ^]^

**Figure 8:**
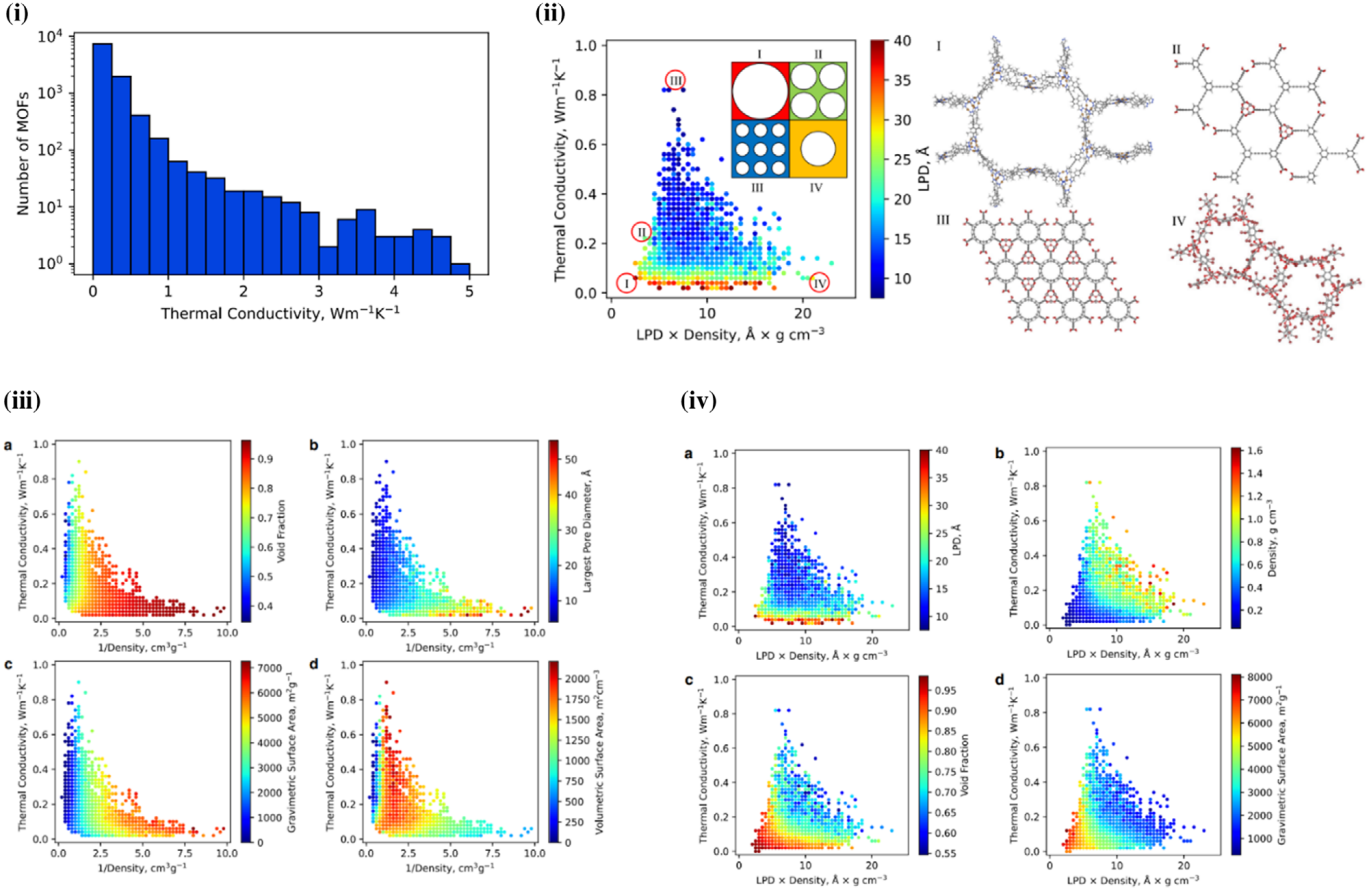
i) Distribution of MOF thermal conductivity data. ii) Hypothetical MOF structures from different regions of the structure‐thermal conductivity space for the relationship between the thermal conductivity and the product of the LPD and density, and their corresponding structures where; I) very low density with very large LPD, II) low density and large LPD, III) high density and small LPD, and IV) high density and large LPD. iii) Relationship between the thermal conductivity and inverse density with a) void fraction, b) largest pore diameter (LPD), c) gravimetric surface area, d) volumetric surface area. iv) Relationship between the thermal conductivity and the product of the LPD and density with a) LPD, b) density, c) void fraction, and d) gravimetric surface area.^[^ ^56^ ^]^ Reprinted with permission.^[^ ^56^ ^]^ Copyright 2023, Nature.

Islamov et al. demonstrated the inherent low thermal conductivity exhibited by MOFs, with >95% of MOFs in this study displaying *κ* < 1 W m^−1^ K^−1^ at 300 K (**Figure** **8**iii). Whilst it was found to be in agreement with the aforementioned studies (i.e., that decreased density, increased void fraction, increased specific surface area and node–linker mass mismatch all contribute to reduced thermal conductivity in MOFs) it was the largest pore size (LPD) that has the most pronounced effect on the thermal conductivity (Figure 8ii,iii). Whilst density, void fraction, and specific surface area are not enough on their own to yield MOFs of ultralow thermal conductivities (<0.5 W m^−1^ K^−1^), sufficiently large pores alone could. In contrast, this is emphasized in the few MOFs of high thermal conductivity (>10 W m^−1^ K^−1^) that were observed during this study–which required a precise combination of structural (pore size, density, surface area, etc.) and constitutional (metal nodes, linker, topology, etc.). As such, this gives us a clear idea that the intrinsic high porosity and low density of MOFs is enough to yield materials of low thermal conductivities, with easy to implement strategies for minimizing *κ* if desired—in contrast to the struggle to achieve high thermal conductivity MOFs.^[^ ^56^ ^]^

Nevertheless, whilst Islamov et al.'s systematic study of MOF thermal conductivity shows clear links in the structure–property relationships, it is not a comprehensive study for TE applications. It only accounts for the lattice contributions to the thermal conductivity (*κ*_l_) and, as such, does not consider the electronic component (*κ*_e_). Neither does it account for the presence of any adsorbates in the MOF pores, as is likely present in experimental scenarios. Adsorbates could be removed via activation, but this may result in the collapse of MOFs with extremely large pores (which are required for the lowest *κ*). Finally, the bond strength between the metal node and linker was also not considered; though, this is likely of minor influence in comparison to the pore size.^[^ ^56^ ^]^

In summary, the high porosity and low density of the majority of MOFs is significant enough to yield low thermal conductivity for TE materials. However, there are also design strategies available to further minimize *κ*_l_. Larger, hexagonal pores should be more efficient at reducing *κ* than smaller pores of other shapes, as too are heavier metals (by increasing the metal–linker mass mismatch). Despite this, to the best of the authors knowledge there are no computational studies on *κ* of intrinsically conductive MOFs, and thus the effect these factors have on *σ* (and as such *κ*_e_) must also be considered when designing TE MOFs.

### 2.2 Electrical Conductivity

Due to their outstanding porosity, the vast majority of MOFs tend to be electrical insulators. As such, a major challenge in the search for TE‐viable MOFs is how to improve their electrical conductivity. Only recently have the electronic properties of MOFs begun to gain traction, and as such, the possibilities have yet to be fully explored.^[^ ^57^ ^]^

The electrical conductivity of a material is dependent on the charge‐carrier mobility, *μ*, and the concentration of charge carriers, *n*, in the material. Understanding how these parameters can be tailored to afford improved electrical conductivity requires an in‐depth understanding of the material's electronic band structure, the charge transport mechanisms occurring, and the interactions of any guest dopants with the host and the charges. To date, these concepts have been largely understudied when it comes to MOFs. Crucially, the origin of electrical conductivity in MOFs has not yet been fully understood. This has also been hindered by the lack of measurements on single‐crystal MOF samples, which would enable the deduction of the intrinsic charge transport mechanism.^[^ ^57^ ^]^

#### 2.2.1 Transport Mechanisms

The transport of charge carriers can typically be described by two main mechanisms: band‐like transport or hopping transport. An understanding of the band structure of an intrinsically conductive MOF could be partly deduced from the observed transport mechanism. Band‐like transport occurs due to strong orbital interactions, which allow charge carriers to be fully delocalized and move through continuous energy bands. This can either be metallic or the charges can be thermally generated—as in a semiconductor. In contrast, hopping transport occurs between sites of localized charge, with the hopping rate determined via a thermally activated barrier.

Typically, electrically conductive MOFs, thus far assessed experimentally, have shown positive dependence of electrical conductivity on temperature. However, it is also important to highlight the limited number of studies on single‐crystal samples, with most having been performed on polycrystalline powder‐pressed pellets. These polycrystalline samples possess many defects and grain boundaries, which could be driving the temperature dependence. One single‐crystal study of Ni_3_(HITP)_2_ displayed a positive Zabrodskii plot indicative of metallic behavior, whilst in contrast polycrystalline film devices displayed semiconducting behavior with a negative Zabrodskii plot. This discrepancy highlights the distinct variation in intrinsic and non‐intrinsic electronic behaviors that have be observed for MOFs thus far, and so to develop a better experimental understanding of their intrinsic transport mechanisms requires more single‐crystal studies.^[^ ^58^ ^]^

**Figure 9:**
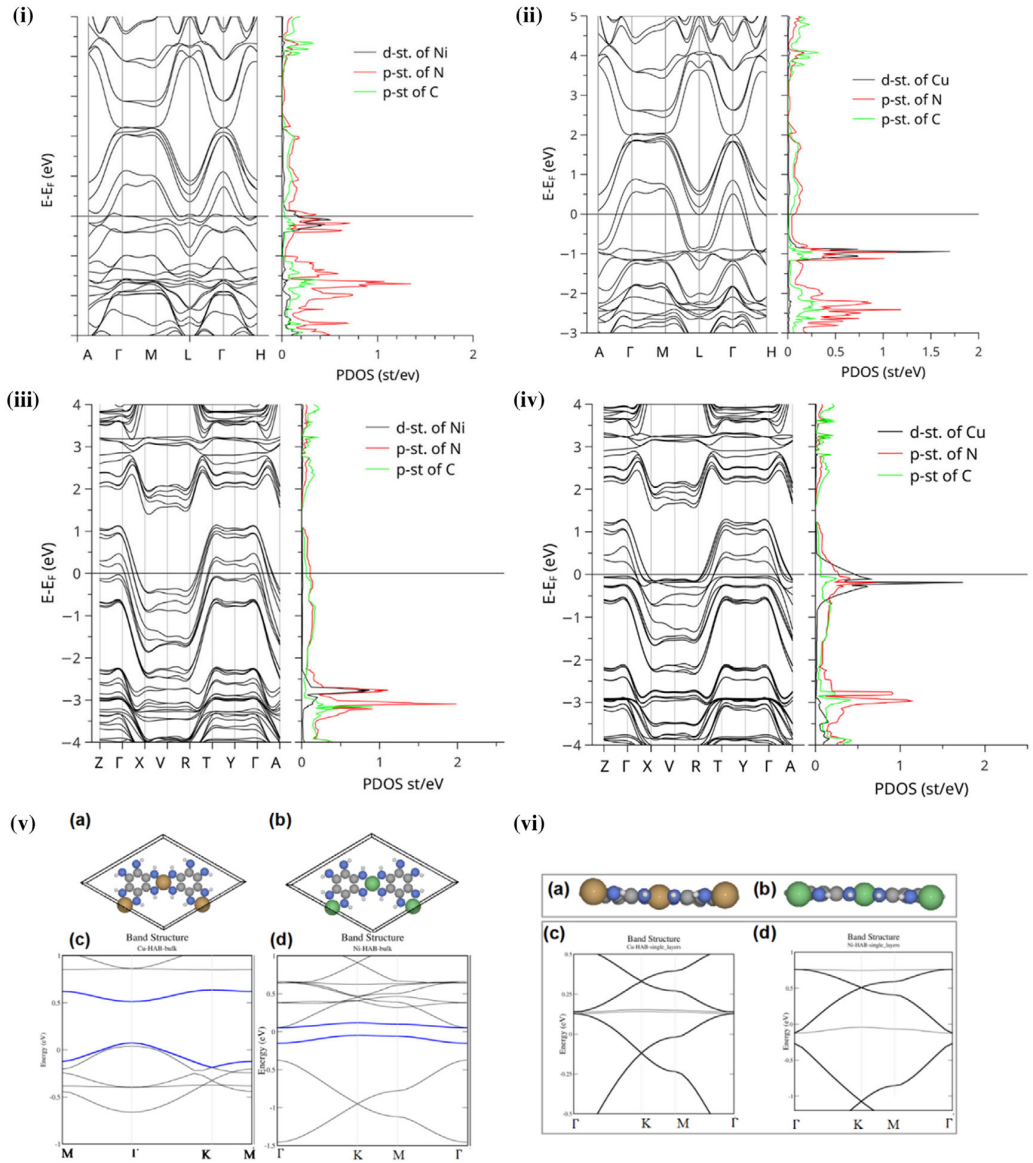
i) Band structure and partial density of states (PDOS) of Ni‐HAB determined via PBE0 functional. ii) Band structure and PDOS of Cu‐HAB determined via PBE0 functional. iii) Band structure and PDOS of Ni_3_(HITP)_2_ bulk determined via hybrid PBE0 functional. iv) Band structure and PDOS of Cu_3_(HITP)_2_ bulk determined via PBE0 functional.^[^ ^59^ ^]^ v) Band structure calculations of bulk (left) Cu‐HAB and (right) Ni‐HAB. vi) Band structure calculations of monolayer (left) Cu‐HAB and (right) Ni‐HAB.^[^ ^60^ ^]^ Reprinted (adapted) with permission.^[^ ^60^ ^]^ Copyright 2018, Springer Nature.

**Figure 10:**
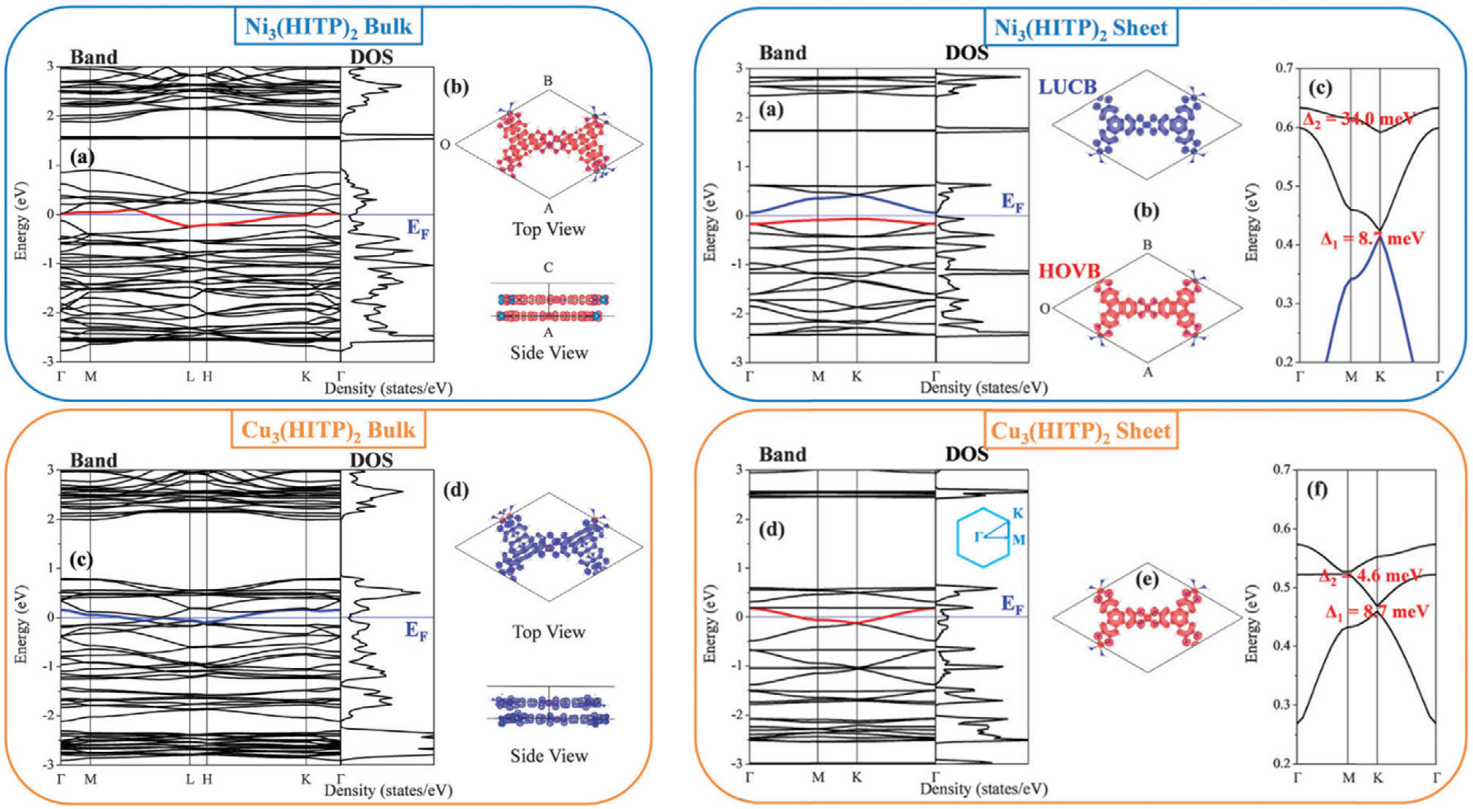
Calculated band structures and PDOS for (top) Ni_3_(HITP)_2_ and (bottom) Cu_3_(HITP)_2_ for bulk (left) and monolayer sheets (right).^[^ ^61^ ^]^ Reproduced with permission.^[^ ^61^ ^]^ Copyright 2018, Royal Society of Chemistry.

Although there have been computational studies accessing the band structures of various electrically conductive MOFs, there persists a lack of consistency between these and experimental data, and even between different computational models. Most of these studies have focused on the most conductive family of 2D planar MOFs (see later)—for which the computational data indicates the significance of the order between the layers in the resulting band structure. While one computational study by Syrotyuk et al. found that the band structure for Ni‐HAB (hexaaminobenzene) and Cu‐HAB MOFs would indicate metallic behavior in the bulk (**Figure** **9**i,ii), another study by Feng et al. disagreed with this, suggesting that while the monolayers of these MOFs were metallic (Figure 9vi), the bulk exhibited a bandgap (Figure 9v).^[^ ^59^, ^60^ ^]^ Likewise, Syrotyuk et al. found the band structures for both bulk Cu_3_(HIITP)_2_ (heximinotriphenylene) and Ni_3_(HITP)_2_ to be semiconductors with a small bandgap (Figure 9iii,iv). Still, other studies by Chen et al. predicted Cu_3_(HITP)_2_ and Ni_3_(HITP)_2_ to be metallic in the bulk, yet only Cu_3_(HITP)_2_ to be metallic in the monolayer whilst Ni_3_(HITP)_2_ monolayer was expected to be semiconducting (**Figure** **10**).^[^ ^61^ ^]^ This highlights the need for improved accuracy in accounting for the input material crystal structure, including defects and expected disorder, for computational models to more accurately reflect what is experimentally observed.

**Figure 11:**
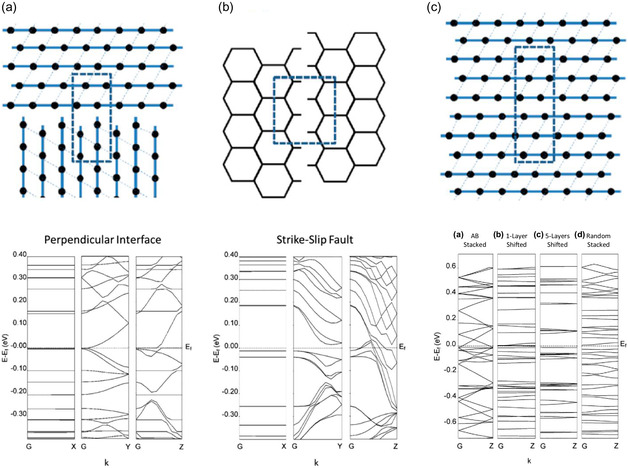
Graphical depiction and corresponding calculated band structures for Ni_3_(HITP)_2_ with a) perpendicular interface, b) strike‐slip fault, and c) lateral layer displacement defects (AB stacked (a), 1‐layer shifted (b), 5‐layers shifted (c), and random stacked (d))^[^ ^62^ ^]^ Reprinted (adapted) with permission.^[^ ^62^ ^]^ Copyright 2018, American Chemical Society.

Foster et al. demonstrated this with a computational study that included the incorporation of three defects: a) perpendicular grain boundary, b) strike‐slip fault between grains, and c) lateral layer–layer displacement (**Figure** **11**).^[^ ^62^ ^]^ These show that the incorporation of defects into the computational model gave rise to bandgaps at the Fermi level of a pristine material Ni_3_(HITP)_2_, giving rise to semiconducting band structures rather than metallic.^[^ ^62^ ^]^ In a separate study, Foster et al. also highlighted the significance of the layer–layer interactions on the band structure of layered MOFs, taking semiconductor monolayer, to metallic bulk back to semiconducting bulk via expansion of layer–layer distance (see later).^[^ ^63^ ^]^

Developing a clear understanding of the origin of intrinsically electrical conductivity in MOFs via their transport mechanisms will be key to designing MOFs of improved *σ*. Currently there appears to be a lack of consistency both between computational methods, and with experimental studies. Ongoing work to develop refined computational methods, including more accurate crystal structure inputs (including defects etc.) will help to achieve this.

#### 2.2.2 Electrically Conductive Pathways in MOFs

**Figure 12:**
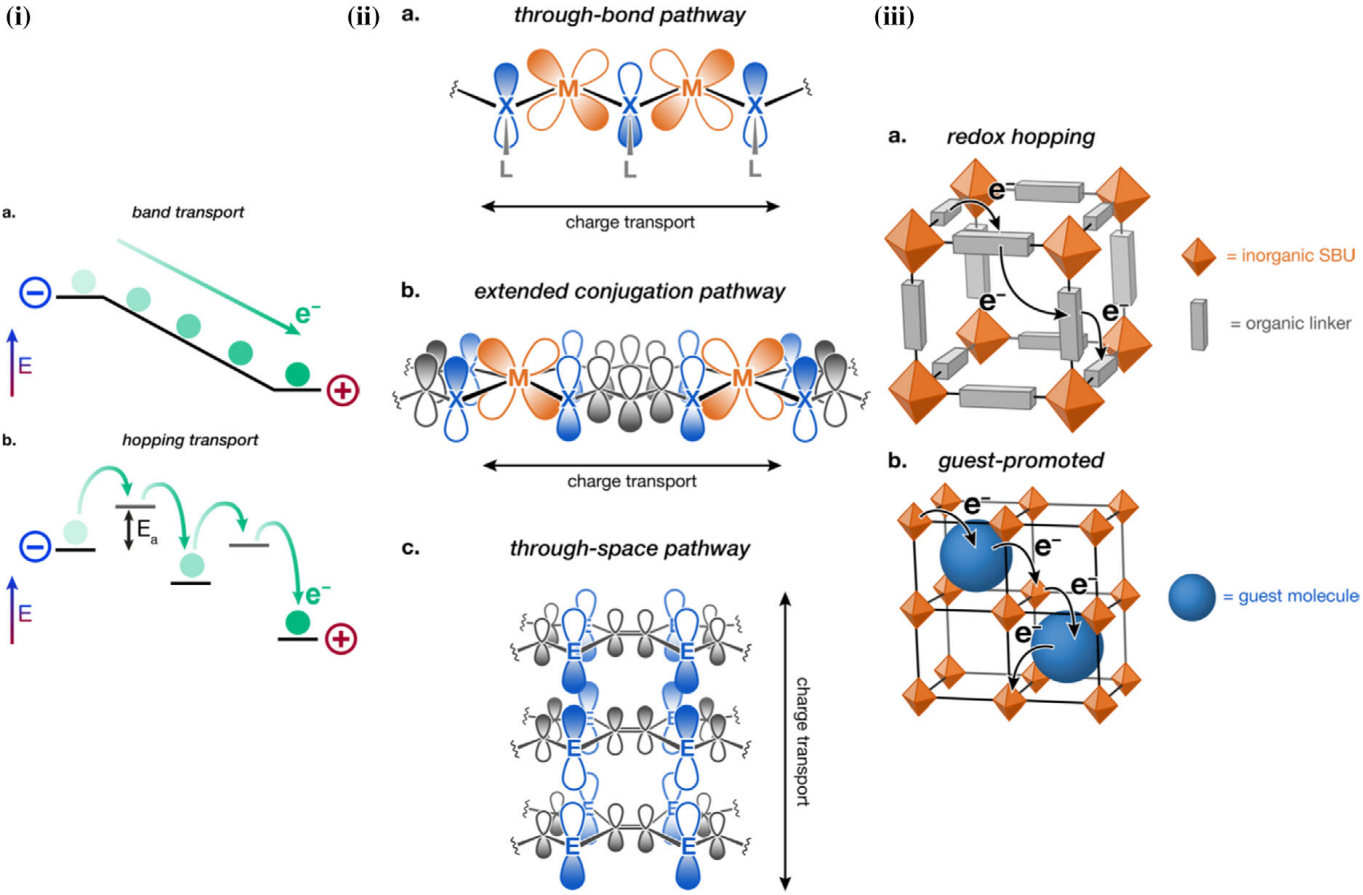
i) Graphical depiction of the difference between band transport and hopping transport mechanisms of electrical conductivity. ii) Orbital representations of charge transport pathways in MOFs; a) “through‐bond” pathways, b) “extended‐conjugation” pathways, and c) “through‐space” pathways. iii) Electrical transport mechanisms that are promoted via redox; a) charge transport via redox hopping between organic moieties; and b) guest‐promoted redox hopping.^[^ ^57^ ^]^ Reprinted (adapted) with permission.^[^ ^57^ ^]^ Copyright 2020, American Chemical Society.

Despite ongoing discussion in the literature of the origin of intrinsic electrical conductivity in MOFs, various conductive MOFs have been successfully designed using three main strategies (**Figure** **12**).^[^ ^57^ ^]^

Firstly, enhancing the through‐bond interactions enhances the conductivity via promoting strong covalent interactions between the orbitals of the metal node and the heteroatom of the organic linker. Typically, most MOFs are synthesized with carboxylic linkages. These tend to introduce a bond of more ionic nature between the metal and the linker, due to the hard‐base nature of O, resulting in larger energy gaps and therefore insulating MOFs.^[^ ^39^ ^]^ In contrast, linkers with softer, less‐electronegative heteroatoms (e.g., S and N) have better energy matching to the lower‐charged transition metals. This results in metal–ligand bonds of more covalent nature, with improved orbital overlap and interactions, giving rise to MOFs with smaller energy gaps.^[^ ^57^ ^]^

Secondly, the employment of ligands that contain an aromatic organic core and chelating functional groups that, upon coordination with the metal, can result in MOFs with 2D conjugated planes. Metal nodes and linkers afford good *π*–*d* interactions and allow the aromatic conjugation to be extended over the whole MOF framework, not just localized to the organic moieties. This will enable effective charge transport through the *ab* plane, of a typically 2D MOF, in an extended‐conjugation pathway.^[^ ^57^ ^]^

Thirdly, the introduction of highly conjugated aromatic planes within the MOFs also allows for a transport pathway via *π*–*π* stacking interactions. This through‐space interaction affords electrical conductivity across the *c* plane of 2D planar MOFs, indicating the significance of the interlayer distance. The smaller the interlayer distance, the larger the charge mobility will be through the *c* plane. Whilst it is suspected that the extended conjugation through the *ab* plane is the major contributor to electrical conductivity in 2D planar MOFs, some computational studies and experimental results have highlighted the likely significant role the through‐space *π*–*π* interactions play.^[^ ^63^, ^64^ ^]^

Overall, these three strategies highlight the significance of the careful selection of the metal‐linker pairing in achieving intrinsic electrical conductivity in MOFs. Good orbital energy matching between the metal cation and linker is paramount in achieving this, both for the through‐bond and extended‐conjugation pathways. This can be achieved by selecting the linker heteroatoms of lower electronegativity (such as sulfur or nitrogen), chelating linkers with aromatic cores, and metals of partially filled *d* orbitals to promote *π*–*d* interactions. Whilst this will help promote good electrical conductivity in the *ab*‐plane, careful tuning of the interlayer distance is also significant for the *π–π* interactions in the *c*‐plane of layered 2D MOFs.

### 2.3 Seebeck Coefficients of MOFs

**Figure 13:**
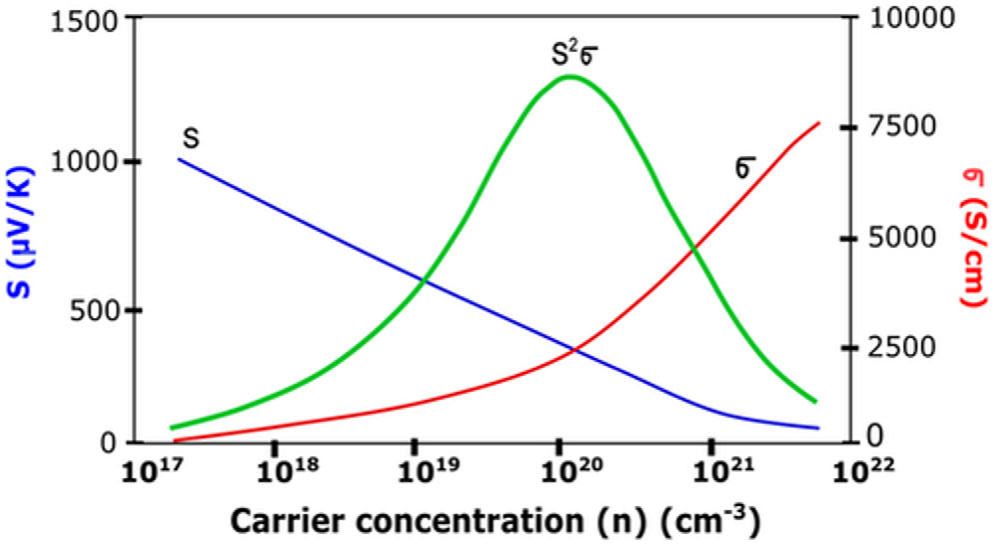
Graph depicting the inter‐dependent relationship between the thermoelectric parameters (*S*, *σ*, and *κ*) and the carrier concentration and how this can result in a carrier concentration for maximized *zT*.^[^ ^108^ ^]^ Reprinted (adapted) with permission.^[^ ^108^ ^]^ Copyright 2019, John Wiley and Sons.

The Seebeck coefficient (thermopower) of a material describes the magnitude of the thermoelectric voltage induced by a temperature gradient across the material. For an effective TE material, it is critical to maximize *S*. From Equation (1), *S* has the most significant influence on *zT* compared to other parameters (*zT* ∝ *S* ^2^). The challenge for enhancing the *S* for a given material arises due to the competing effects of the other parameters (*σ* and *κ*) that influence *zT*. The electrical conductivity can be improved by increasing the charge‐carrier concentration, *n*; however, this will result in a decrease in the Seebeck coefficient (**Figure** **13**). As such, there is an optimum charge‐carrier concentration for a maximum power factor (PF = *S* ^2^ *σ*).^[^ ^43^ ^]^

The band structure of a material and the relative position of its Fermi level significantly influence the magnitude of the Seebeck coefficient, and so understanding of the material's band structure is vital for optimizing *S*. For metals, where the Fermi level resides within a band, *S* is, therefore, low in magnitude despite their typically high electrical conductivities. Intrinsic semiconductors with Fermi levels within the band gap can yield *S* of large magnitude due to the breaking of symmetry between the electrons and holes as a result of a band gap. However, intrinsic semiconductors are typically limited by lower electrical conductivity. As semiconductors are doped, the Fermi level is moved closer to the band edge thus increasing the availability of electronic states for improved electronic conductivity. In the low doping regime, *S* is proportional to the energy difference between the Fermi level and the transport level, so doping will reduce *S*. On the contrary, in the heavily doped regime the Mott relationship applies and the Seebeck coefficient is proportional to the rate of change of the logarithm of conductivity with energy at the Fermi level $\left({\frac{d\left(\ln\sigma \right)}{dE}|}_{E_{F}}\right)$.^[^ ^65^ ^]^ In this case, the Seebeck coefficient is maximized when the Fermi level is near to the edge of the conduction band. Consequently, to achieve high *S* while maintaining high *σ*, low bandgap semiconductors, or heavily doped semiconductors are required.

An observable disadvantage of TE MOFs so far tested (Table 1) is the relatively low *S* values, and, as such, despite the relatively good electrical conductivity values so far reported, *zT* is limited. Such low *S* values are an indication of these MOFs having metallic character, rather than the more desirable heavily doped semiconductor. The metallic versus semiconducting nature of these MOFs has been an area of discussion within the field. Whilst experimentally many of these MOFs have been suggested to exhibit a positive temperature dependence on the electrical conductivity, many computational studies of their band structures have predicted them to be metallic.^[^ ^29^, ^38^, ^40^, ^41^, ^42^, ^48^, ^63^, ^66^, ^67^ ^]^ However, a study of Ni_3_(HITP)_2_ by Foster et al. demonstrated that stacking defects introduced into the MOF structure resulted in the appearance of a bandgap, from what had previously been a metallic band structure. This highlights one way in which synthesized MOFs may have different charge transport properties to computational predictions of defect‐free single crystals.^[^ ^62^ ^]^

**Figure 14:**
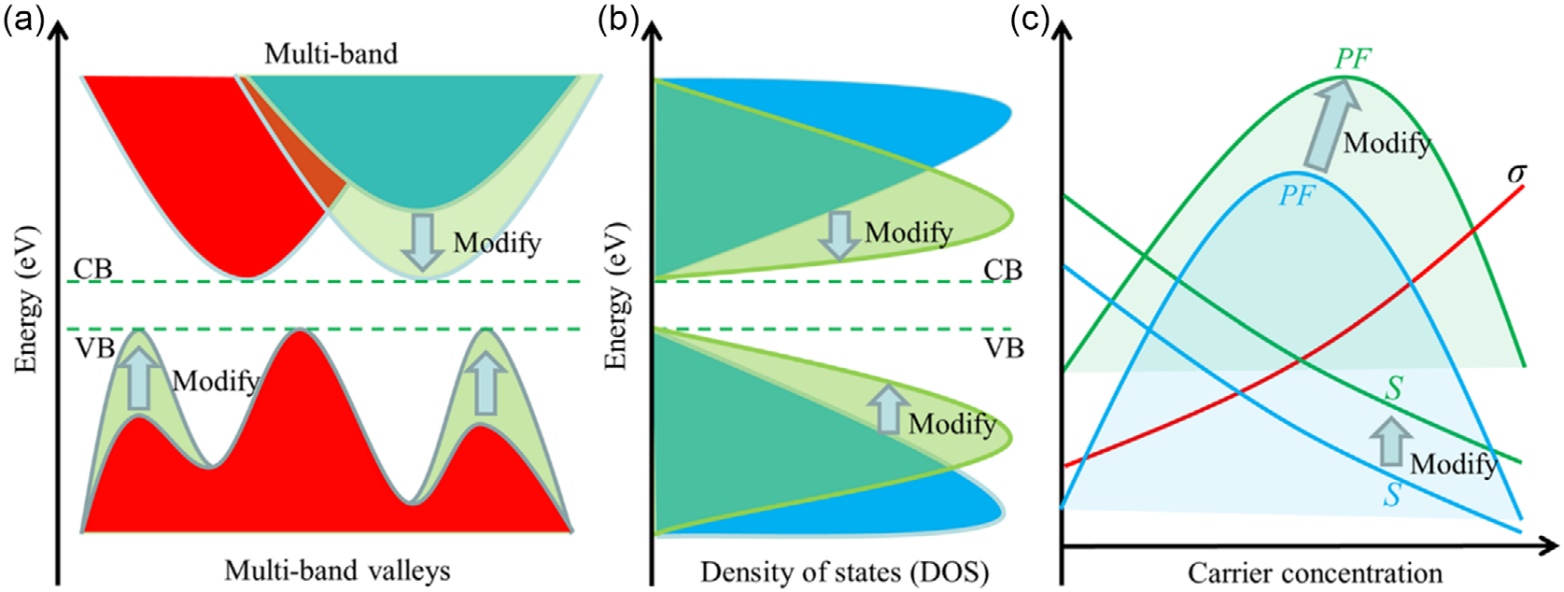
Electronic band structure engineering demonstrated for layered oxyselenides resulting in the modification of the a) band valleys, b) density of states, and c) the effect on the *PF* and *S*.^[^ ^69^ ^]^ Reprinted with permission.^[^ ^69^ ^]^ Copyright 2019, Nature.

Single‐crystal studies will aid understanding of the origin of the intrinsic conductivity within these MOFs and their electronic band structures. Having a robust understanding of a materials electronic band structure is vital for utilizing the structure–property relationship to design MOFs with optimal *S* and therefore *ZT* values. Effective band engineering can boost *S* via band degeneracy. Band degeneracy refers to the scenario where multiple bands can be found at the same energy level (within a few *k*_B_ *T*).^[^ ^68^ ^]^ This can be particularly prominent in layered materials (i.e., highly conductive 2D layered MOFs mentioned later), and as such layered materials are of great interest for TE. Wang et al. demonstrated the possibility of boosting PF performance via modification of the band structure of layered oxyselenides to enhance *S*, (**Figure** **14**).^[^ ^69^ ^]^ Effective understanding of the band structures of, particularly layered, MOFs will also allow for future development toward materials with engineered band structures for improvement of TE properties.

To summarize, optimizing of the *S* is crucial for enhancing the performance of TE MOFs, due to the significant impact on *zT*. Typically, the *S* of intrinsically conductive MOFs reported thus far have been low, owing potentially to their metallic band structure. Better understanding of the intrinsically conductive MOF band structure is required to better design MOFs of the ideal heavily doped semiconductors to maximize *S* whilst maintaining high *σ*. This can be achieved both with improvements in computational models and single‐crystal studies. In depth understanding of the MOF band structures could also allow utilization of band engineering in the design process, which could be especially impactful for 2D layered MOFs in improving the *S*.

### 2.4 Current MOFs with Reported Thermoelectric Properties

The emergence of intrinsically conducting MOFs has slowly been gaining traction; however, their application as TE materials remains greatly understudied. In Table 1, we summarize MOFs evaluated so far for TE properties. Even though MOFs are expected to have ultralow thermal conductivities, typically with values <0.4 W m^−1^ K^−1^, very few MOFs that have been tested for TE properties (*S* and *σ*) have also had their thermal conductivity reported.^[^ ^43^ ^]^ Whilst the thermal conductivities are expected to be low, their lack of reported measurements has also meant that very few MOFs have *zT* values reported. This is a major drawback in the current field of TE MOFs, which prevents the comparison of *zT* values between MOFs and current state‐of‐the‐art TE materials.

**Figure 15:**
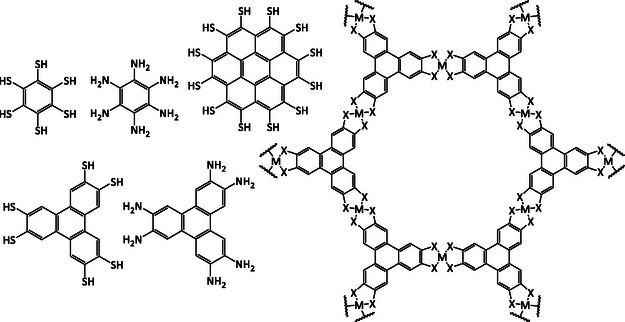
(Left) Chemical structure of HXTP and HXB linkers: HTB, HIB, HTTP, HITP, and PTC. (Right) Structural framework of HXTP MOF in *ab* plane, which extends into honeycomb sheets that stack in layers in *c* plane. Where X = O, S or NH.

Currently, the vast majority of MOFs that display intrinsic conductivity, and have been assessed for their TE behavior, are 2D planar based of the same family of ligands (HXTP and HXB, where X = O, S, or NH), formed with square planar coordination around the metal center, (**Figure** **15**).^[^ ^38^, ^41^, ^42^, ^47^, ^70^, ^71^, ^72^ ^]^ This series has consistently demonstrated the highest electrical conductivities in MOFs so far, thus making them good initial candidates for TE materials.^[^ ^41^ ^]^

The exceptions to these are Zn_3_(HIB)_2_, which, whilst formed with the same HIB linker, has a tetrahedral microenvironment around the Zn center and, as such, results in a 3D structure, and Ni_3_(PTC), which is based on a perthiolated‐coronene (PTC) linker with a larger aromatic core compared to HXTP and HXB.^[^ ^40^, ^48^ ^]^ Nonetheless, there is clearly a lack of compositional diversity amongst the TE MOFs evaluated so far. Given the structural diversity the organic moieties could provide, multiple routes to fine‐tune and enhance the TE properties could be explored.

Whilst other 3D MOFs have been assessed for TE properties, these have all involved the incorporation of guest molecules into the pores. Zn‐HIB is, therefore, the only intrinsically conductive 3D MOF tested thus far to the best of the authors’ knowledge.^[^ ^27^, ^39^, ^48^ ^]^ The benefits of a 3D MOF for TE are the better ability to incorporate guest molecules into the pores for doping to enhance the TE properties, and for more significant phonon scattering, resulting in lower thermal conductivities; however, neither of these have yet been demonstrated for this Zn‐MOF. As such, we are unable to compare its full potential *zT* value to 2D MOFs. In effect, whilst Zn‐HIB did display electrical conductivities of 0.86 × 10^−3^ S cm^−1^, this was significantly lower than the conductivities reported for the 2D MOFs. This could be attributed to the loss of the extended conjugation and through‐space pathways due to the 3D nature of the MOF.^[^ ^48^, ^57^ ^]^ It could also be due to more localized orbital interactions between the Zn and HAB linker, compared with other MOFs—as demonstrated by the difficulty during synthesis. This lack of delocalization should be evident in the Zn MOF band structure, resulting in a larger bandgap supported by the relatively large *S*.^[^ ^48^ ^]^

**Figure 16:**
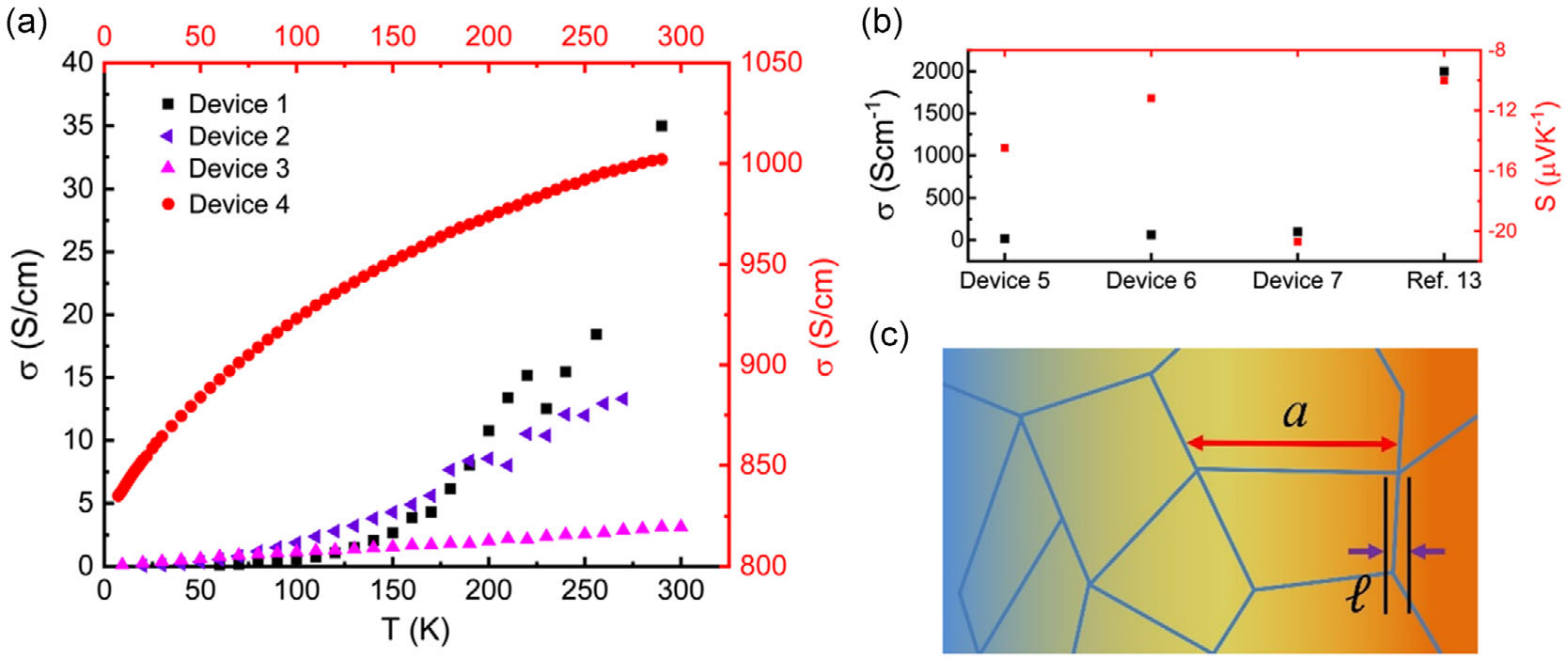
a) The difference in temperature dependence electrical conductivity measurements for devices of Cu‐BHT; positive temperature dependence in Device 1–3 (left axis), and weaker positive temperature dependence in Device 4 (right axis). b) Electrical conductivity and Seebeck measurements at room temperature for Cu‐BHT devices 5–7 compared to a reference sample. c) Illustration demonstrating how the temperature gradient (denoted by the color gradient) is larger over the size of a crystalline domain (*a*) compared to the size of the boundary (*l*).^[^ ^41^ ^]^ Reprinted (adapted) with permission.^[^ ^41^ ^]^ Copyright 2020, John Wiley and Sons.

Currently, the largest reported *zT* value (0.013) for an intrinsically conductive MOF has been measured for to Cu_3_(HIB)_2_ (**Figure** **16**).^[^ ^41^ ^]^ However, this is still significantly lower than that reported for the current state‐of‐the‐art inorganic TE materials (e.g., for alloys of Bi_2_Te_3_, *zT* > 1).^[^ ^12^, ^15^, ^43^, ^73^ ^]^ The high *zT* value of Cu_3_(HIB)_2_ is attributed to its exceptionally high *σ* (2000 S cm^−1^), the highest reported for any intrinsically conductive MOF so far. Cu_3_(HIB)_2_ has consistently demonstrated high electrical conductivities, with another study reporting values up to 1580 S cm^−1^.^[^ ^47^ ^]^ Despite this, not all devices fabricated from this MOF displayed such high *σ*; as low as 3 S cm^−1^ was also reported in the same study.^[^ ^41^ ^]^ The low performance was attributed to variations in the degree of polycrystallinity of the samples measured, rather than differences in sample aging or doping. This was determined from the differences in how the samples’ electrical conductivity varied with temperature, Figure 16.^[^ ^41^ ^]^

This exceptionally high electrical conductivity of Cu_3_(HIB)_2_, combined with a denser MOF structure with smaller pores, compared to HXTP analogs, results in a relatively higher thermal conductivity (1.99 W m^−1^ K^−1^). This, coupled with the relatively low *S* (−21 μV K^−1^), a value more typical of metals, limits the *zT*. In fact, the low *S* value could suggest the presence of crystalline metallic domains separated by grain boundaries.^[^ ^41^ ^]^

It should also be noted that this *zT* value for Cu_3_(HIB)_2_ was estimated from a combination of the best results reported for *σ* (Figure 16; device 4), *κ*, and *S* (Figure 16; device 7).^[^ ^41^ ^]^ As such, it is an overestimation of *zT* and does not account for the inter‐competing nature of these parameters. Nevertheless, Cu_3_(HIB)_2_ represents a promising starting point for the development of TE MOFs.

As previously mentioned, there have been limited experimental studies on the thermal conductivity of these MOFs. As such, only three MOFs have *zT* values reported to date. However, all MOFs are expected to have relatively low thermal conductivities and so their PF should give a good indication of the material's TE performance. Cu_3_(HIB)_2_ still reports the highest PF for MOFs so far (0.88 μW cm^−1^ K^−2^), compared to other MOFs for which the PF is up to five orders of magnitude smaller. Again, this is due to the high electrical conductivity of Cu_3_(HIB)_2_, and whilst other MOFs display larger *S* values (up to −340 μV K^−1^) they are greatly hindered by their inferior electrical conductivities.

Considering the high modularity, recent developments, and lack of systematic investigations, it is clear that there is plenty of room for the design and optimization of new MOFs tailored toward high‐ performance TE materials. Currently the field is limited by the lack of understanding of the MOFs’ band structures, with most MOFs thus far demonstrating low *S* values, more typical of metals. In addition to these low *S* values, the MOFs are likewise limited by the relatively low electrical conductivities, except for a few. Band engineering by chemical design should therefore be at the heart of future strategies to improve TE performance in MOFs.^[^ ^74^, ^75^ ^]^ This will require in‐depth understanding of the materials’ structure–function relationship to design TE MOFs with appropriate band structures for optimized TE properties, including careful selection of metals and linkers.

## 3 Design Strategies of MOFs toward Efficient Thermoelectric Materials

Despite the wide structural diversity of MOFs, with over 100 000 different structures reported to date and a further 400 000 structures predicted—so far, the search for thermoelectric MOF materials has been limited to mostly one topology. As such, there is a chance that better‐performing MOFs have already been synthesized.

A unique property and expected benefit of MOFs lies in their structural diversity, arising from the organic linkers, yet this has been underused in the development of TE MOFs. Developing an understanding of the origins of the TE properties in MOFs can aid in the strategic design of new linkers for new MOFs tailored toward improved TE behaviors. Machine learning based on an expanding database of MOF electronic and crystal structures will be important in accelerating the search for improved TE MOFs.

A challenge in the optimization of intrinsically conductive MOFs for application as TE materials leads from the challenges surrounding the computational studies of the MOFs’ electronic properties and band structures. The extensive and complicated crystal structures of MOFs can make computational studies via DFT computationally expensive and time consuming. The development of tailored computational programs for the study of electronic and TE properties of MOFs will help with achieving accurate theoretical understanding of the origins of the electronic properties and band structures more easily—allowing for advancement toward higher performance TE MOFs.

**Figure 17:**
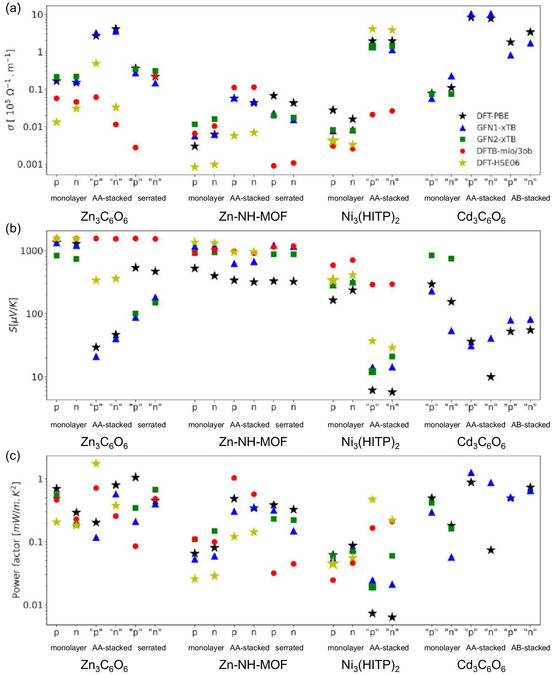
The maximum TE properties a) electrical conductivity, b) Seebeck coefficient, and c) PF) computationally determined via four different computational methods, DFT‐PBE (black stars), GFN1‐xTB (blue triangle), GFN2‐xTB (green square), DFTB‐mio/3ob (red circle) and DFT‐HSE06 (yellow star), for the Zn_3_C_6_O_6_, Zn‐NH‐MOF, Ni_3_(HITP), and Cd_3_C_6_O_6_ MOF systems.^[^ ^76^ ^]^ Reprinted (adapted) with permission.^[^ ^76^ ^]^ Copyright 2024, American Chemical Society.

An extensive computational study by Gahrouei et al. highlighted the challenges of the methodology discrepancies between a series of computational methods (DFT‐PBE, GFN1‐xTB, GFN2‐xTB, DFTB‐mio/3ob, and DFT‐HSE06) in predicting the electronic and TE properties of a series of MOFs (**Figure** **17**).^[^ ^76^ ^]^ Differences in the predicted band structures and band gaps between the MOFs impacted the predicted TE properties; DFT‐PBE predicted smaller bandgaps (and hence higher electrical conductivities but lower S) compared to DFT‐HSE06 and DFTB‐mio/3ob. Improving and developing computational models that are less computationally expensive will aid in developing the improved understanding of intrinsically conductive MOFs’ electronic properties required for the strategic development of MOFs tailored for TE applications.^[^ ^76^ ^]^

The next sections will discuss the effects of varying different structural aspects of intrinsically conductive MOFs. Whilst TE MOFs is an emerging field with few studies, electrically conductive MOFs have a wider application potential in a variety of fields and as such have attracted more research interest. As MOF design with intrinsic electrical conductivity is one of the major challenges in the development of MOFs as TE materials, this section will mostly focus on this, and how it could, in turn, influence the overall TE properties.

### 3.1 Metal Substitution

**Figure 18:**
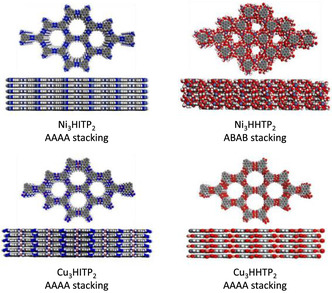
Space‐filling models for Ni_3_(HITP)_2_, Ni_3_(HHTP)_2_, Cu_3_(HITP)_2_, and Cu_3_(HITP)_2_ MOFs showing part of the extended framework and changes in predicted stacking patterns with different heteroatoms.^[^ ^90^ ^]^ Reprinted (adapted) with permission.^[^ ^90^ ^]^ Copyright 2020, American Chemical Society.

To produce the 2D planar structures desired for extended conjugation in HXTP and HXB‐based MOFs, metal nodes with a preference for square planar geometry were selected. The consensus for these frameworks has been that the substitution of different metal ions with preference for square planar geometries, will yield MOFs that are isostructural to one another.^[^ ^31^, ^72^ ^]^ As such the structure of the MOFs should be insensitive to different metals, allowing for tuning of chemical and electronic properties by varying metals. Whilst this structure agnosticity has mostly been the case, there are minor exceptions, such as, slight changes in the stacking arrangements and interlayer distances, **Figure** **18**.

The synthesis of conductive MOFs with different metal nodes would therefore offer the ability to tailor the MOFs’ chemical and electronic properties without necessarily impacting the framework structure. As such, a range of HXTP and HXB MOFs have been reported with different metals, aiming to achieve high electrical conductivities. Developing a better understanding of the effect of varying metal ions will help with the development of new MOFs tailored for TE applications.

#### 3.1.1 Metal Node Topology

These 2D conductive MOFs are generally considered isostructural to one another. However, a computational study by Chen et al. indicated that changing from Ni to Cu in M_3_(HITP)_2_ results in a buckling of the planar MOF at the metal site.^[^ ^61^ ^]^ Ni is predicted to adopt a “perfect square planar” geometry, due to its *d* ^*8*^ electron count, with *dsp* ^*2*^ hybridization resulting in perfect conjugation across the 2D sheet. In contrast, Cu (II) *d* ^9^ is predicted to be *sp* ^3^ hybridized at the Cu center, which results in the above‐mentioned buckling of the 2D sheet—potentially disrupting the conjugation across the 2D layer.^[^ ^61^ ^]^ This could also account for the higher conductivity reported for Ni_3_(HITP)_2_ compared to Cu_3_(HITP)_2_ in a separate study by Chen et al. (55 and 0.75 S cm^−1^, respectively).^[^ ^64^ ^]^

Such change in configuration was also noted between Co_3_(HITP)_2_ and Ni_3_(HITP)_2_ by Lian et al. Whilst the Ni center has D_4h_ symmetry, this symmetry is broken for Co due to an unpaired electron in its *d* ^7^ configuration, resulting in a Jahn–Teller effect distortion. This destabilizes the perfect square planar geometry, disrupting the extended conjugation.^[^ ^64^, ^77^ ^]^

Hinckley et al. performed a systematic study of the effect of metal substitution on the electronic properties of M_3_(HIB)_2_ MOFs.^[^ ^72^ ^]^ The results indicated that Co_3_(HIB)_2_ had the highest conductivity in inert atmosphere, but it decreased in air, whilst Ni_3_(HIB)_3_ (second‐highest conductivity) remained constant; however, both were considerably higher than Cu_3_(HIB)_2_. The differences in conductivities were attributed to the changes in *d* electron count from metal to metal. From a square planar crystal field splitting, the highest and lowest *d* orbitals were assigned as $d_{xy}$ and $d_{x^{2}-y^{2}}$. As such, the highest occupied *d* orbital for each metal: Co^2+^ *d* ^7^ and Ni^2+^ *d* ^8^ would have significant $d_{z}$ character, whereas Cu^2+^ (*d* ^9^) would be $d_{x^{2}-y^{2}}$. The *d* orbitals with more *z*‐character have metal orbitals that are able to interact and partake in the extended conjugation across the MOF. This means that the $d_{z}$ orbitals of the metal have considerable influence on both the extended‐conjugation and through‐space charge‐carrier pathways. Consequently, Hinckley et al. suggested that the higher electrical conductivity of Co and Ni compared to Cu is due to the highest occupied *d* orbital in both having significant *z* character.^[^ ^72^ ^]^ In contrast, for HTB MOFs, Cu is consistently reported to have the largest electrical conductivity, indicating that the linker heteroatom must also play a significant role in the metal preference, and it likely has a more complex influence—see discussion later.^[^ ^41^ ^]^

**Figure 19:**
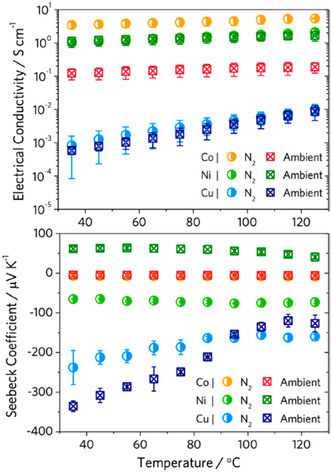
Temperature‐dependent electrical conductivity (top) and Seebeck Coefficient (bottom) measurements for HIB MOFs (Co, Ni, and Cu) in N_2_ and ambient atmosphere.^[^ ^72^ ^]^ Reprinted (adapted) with permission.^[^ ^72^ ^]^ Copyright 2020, American Chemical Society.

Furthermore, Hinckley et al. also demonstrated the significance of the measurement conditions for three HIB‐based MOFs (Co, Ni, and Cu).^[^ ^72^ ^]^ The electrical conductivities and Seebeck coefficients for these MOFs were recorded in both inert (N_2_) and ambient atmospheres. Whilst only the Co MOF demonstrated a significant change in electrical conductivities (a decrease of ≈97% from inert to ambient atmosphere), there were significant changes in *S* observed for all MOFs (**Figure** **19**).^[^ ^72^ ^]^ Under inert conditions, all three MOFs exhibited negative *S* values, indicating that electrons were the majority charge carriers. Co_3_(HIB)_2_ showed the smallest *S* values, likely indicating metallic character, and this decreased further in ambient conditions. The *S* for Cu_3_(HIB)_2_ was the largest in inert conditions (−240 μV K^−1^), and this increased further in ambient conditions to yield a *S* of −340 μV K^−1^; this was also the largest reported *S* for a conductive MOF.^[^ ^72^ ^]^ This huge increase in magnitude was attributed to p‐type doping in an n‐type material. Despite these large *S* values reported, the PF value for each of these MOFs is still significantly smaller than Cu_3_(HTB)_2_ due to the limitation of their electrical conductivities.

Similar to Cu, Ni_3_(HIB)_2_ also demonstrated a significant change in *S* between inert and ambient conditions, attributed to p‐type doping. However, the doping in the Ni MOF appears to be more extensive and results in a total charge‐carrier population inversion, with the sign of *S* changing from negative to positive. This is in line with reports that Ni_3_(HITP)_2_ can behave as an ambipolar material, having displayed both negative and positive *S* values.^[^ ^30^ ^]^ Temperature‐dependent studies for these materials indicated that the origin of the p‐type doping under ambient conditions was due to oxygen physiosorbed onto the surface of the MOF. As temperatures increased above 350 K, the doping was destabilized as fewer O_2_ molecules were absorbed to the surface, trapping n‐type states. This physisorption doping is further confirmed, as heating the MOFs under N_2_ restored their properties to alike under inert atmosphere. This demonstrates how sensitive the electrical properties are to the sample environment. It shows that much care should be taken when measuring and reporting, it also highlights the potential to manipulate conditions to enhance TE properties.

While the interchange of metal cations (Ni, Co, and Cu) can yield isostructural MOFs, variations in their electronic structures can cause minor structural impacts, such as buckling of planar sheets. These alterations significantly affect charge‐carrier pathways, leading to notable differences in electrical conductivities. Although this does not fully explain changes in the Seebeck coefficient and the varying impact of atmospheric adsorbates, further in‐depth computational studies could enhance our understanding of how metal substitution influences band structure and, consequently, the thermoelectric properties of MOFs.

#### 3.1.2 Metal Size

He et al. performed a computational study on Ni, Pd, and Pt HITP MOFs.^[^ ^42^ ^]^ Their predicted band structures indicated that changing the metal influenced which orbitals contributed to the valence band maximum (VBM) and conduction band minimum (CBM). Going down the group (Ni, Pd, Pt) increased the number of metal orbitals that contributed to both the CBM and the VBM. Whilst the electronic band structure shape for the conduction band (CB) remained mostly constant, with changing metals, there were significant changes in the valence band (VB) shapes. This is likely due to the change from the ligand‐orbital dominated VB in Ni‐HITP, compared to the metal‐orbital dominated VB in the Pd and Pt MOFs. Going down the group (Ni, Pd, Pt), the valence *d* orbitals of the metal become larger and more disperse, allowing for better orbital overlap between the ligand and the metal. Consequently, going down the group results in a stronger interaction between the metal and the heteroatom of the linker, and thus results in band structures with more dispersion. He et al. used this to predict that down the group the HITP MOFs would exhibit a larger bandgap and hence larger *S* values. Yoon et al. confirmed this experimentally (Table 1), with the magnitude of *S* increasing when moving to larger metals; Ni (−13.4 μV K^−1^), Pd (19.6 μV K^−1^) and Pt (37.7 μV K^−1^).^[^ ^70^ ^]^ Interestingly, this also confirmed the predicted switching from n*‐* to p‐type semiconductors going down this group.

Typically, a large *S* would be associated with a small intrinsic electrical conductivity. However, in this computational study both the *S* and electrical conductivity were predicted to increase with increasing metal size. This could be attributed to the improved metal‐linker orbital interactions from the larger more disperse metal orbitals improving the “through‐bond” conductivity pathways as well as the previously mentioned band structure dispersion. This is again supported by Yoon et al. who observed small gradual increases in the electrical conductivities with increasing metal size; Ni (0.195 S cm^−1^), Pd (0.261 S cm^−1^), and Pt (0.327 S cm^−1^), measured via two‐probe method which is likely to underestimate contact resistance in the measurement. This contact resistance underestimation may explain why other studies have reported electrical conductivities for the Ni_3_(HITP)_2_ MOF that are significantly greater (58.8 S cm^−1^) or the difference may be due to structural disorder (as illustrated by larger reported interlayer distance of <4.1 Å for the low conductivity material compared to 3.3 Å for the high conductivity material), which could limit the intrinsic electrical conductivity.^[^ ^38^, ^70^ ^]^ The higher conductivities of the Pd and Pt MOFs is also despite of them both being reported as amorphous, which would be expected to yield lower conductivities. As such, achieving high‐quality crystalline samples of Pd and Pt HITP‐based MOFs could achieve even higher electrical conductivities when measured by more precise methods (e.g., four‐probe or van der Pauw).^[^ ^70^ ^]^

Group 11 metals (Cu, Ag, and Au) have all been incorporated in HTB MOFs.^[^ ^41^, ^47^, ^78^, ^79^ ^]^ With Cu_3_(HTB)_2_ being a record‐holder MOF in terms of intrinsic electrical conductivity^[^ ^41^, ^79^ ^]^ and assuming these MOFs might followed a similar trend as the group 10 metals, Au_3_(HTB)_2_ and Ag_3_(HTB)_2_ could offer great potential for high performance TE materials.^[^ ^42^, ^70^ ^]^ Whilst Ag_3_(HTB)_2_ was successfully synthesized, with high electrical conductivities of 363 S cm^−1^, Au_3_(HTB)_2_ was described as insulating (1.12 ×10^−4^ S cm^−1^), attributed to its very poor crystallinity—resulting in random orientation of MOF layers lacking both planar conjugation (disrupting through‐bond and extended‐conjugation pathways) and *π*–*π* stacking (disrupting through‐space pathway).^[^ ^78^ ^]^ However, investigations into optimizing Ag and Au HTB MOF synthesis could yield materials with higher electrical conductivities. It should however be noted that unlike Cu_2_(HTB)_3_, the Ag and Au analogs are in the +1 oxidation state, compared to Cu (II), and so might be unable to display the same significant improvements as predicted for the aforementioned Group 10 metals.

Thus far, these intrinsically conductive 2D MOFs have mostly been restricted to the first‐row transition metals. Despite this, both He et al. and Yoon et al. have demonstrated the significant potential of utilizing larger metal cations to achieve improved metal‐linker orbital interactions for both increased electrical conductivities and *S*, despite lack of high‐quality crystalline samples.^[^ ^42^, ^70^ ^]^ Nevertheless, further insight can be gained on sufficient progress in synthesizing highly crystalline samples.

#### 3.1.3 Mixed‐Valence Metals

Mixed valency in compounds is widely accepted as a means to afford interesting electrochemical properties. Intervalence charge transfer (IVCT) in molecular mixed‐valence complexes has resulted in semiconductors with high electrical conductivities.^[^ ^57^, ^80^ ^]^ This mixed valency can offer electrical conductivity pathways via redox hopping through a MOF framework. The Fe(II)/Fe(III) redox couple is often exploited in mixed‐valence systems, due to the low reduction potential, and minimal changes to coordination spheres.^[^ ^57^ ^]^ As such, Fe_3_(HTTP)_2_, formed with this redox couple, exhibits the highest reported electrical conductivity for HTTP‐based MOFs.^[^ ^24^ ^]^ This is despite of the presence of NH_4_^+^ counterions (see discussion later). The second‐highest electrical conductivity for HTTP MOFs also featured a mixed‐valence Co(II)/Co(III) redox couple, however the HTTP MOFs have further room for optimization including complete oxidation of the MOFs to yield charge‐neutral frameworks.^[^ ^81^ ^]^

Unfortunately, despite the possibilities afforded by mixed‐valence MOFs, the majority of HXTP and HXB MOFs do not exhibit mixed valency, and instead typically feature the metal solely in its +2 oxidation state. This is due to the majority of the redox activity, within these MOFs, being centered on the organic moieties instead.^[^ ^82^ ^]^ The presence of mixed valency in some MOFs was also attributed to undesired side reactions.^[^ ^70^, ^78^, ^83^, ^84^ ^]^ This was particularly problematic for noble metals (Pt, Pd, Ag, and Au), which due to their positive reduction potentials, resulted in the formation of metal nanoparticles, and amorphous MOFs with poor crystallinity.^[^ ^70^, ^78^, ^83^, ^84^ ^]^ However, the potential of redox doping post synthesis could offer the ability to exploit mix valency in these frameworks. It therefore appears that mixed valency may not lead to the desired outcome for TE MOFS.

#### 3.1.4 Bi‐Metallic MOFs

**Figure 20:**
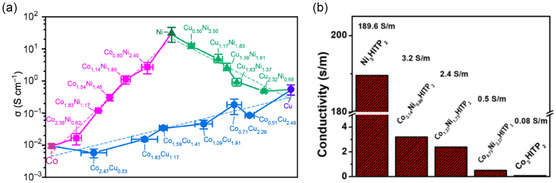
The continuous variation in electrical conductivity as a function of bi‐metallic metal ratios in a) HITP MOFs (Ni, Co, and Cu) and b) HITP MOFs (Ni and Co).^[^ ^34^, ^64^ ^]^ Reprinted (adapted) with permission.^[^ ^64^ ^]^ Copyright 2020, American Chemical Society. Reprinted (adapted) with permission.^[^ ^34^ ^]^ Copyright 2020, John Wiley and Sons.

Doping semiconductors with elements with higher (n‐type) or lower (p‐type) valency has been effectively utilized for many years to enhance electrical conductivity. Conversely, the synthesis of MOFs with different proportions of metals did not lead to improved electrical conductivity.^[^ ^34^, ^64^ ^]^ Rather, Chen et al. demonstrated the ability to have continuous variation in electrical conductivity by changing the proportions of metals in HITP‐based MOFs (**Figure** **20**a).^[^ ^64^ ^]^ Mixed‐metal MOFs (Co_*x*_Ni_3−*x*_(HITP)_2_, Co_*x*_Cu_3−*x*_(HITP)_2_, and Cu_*x*_Ni_3−*x*_(HITP)_2_) were successfully synthesized with uniform dispersion confirmed via STEM elemental mapping. Ni_3_(HITP)_2_ exhibited the highest conductivity, compared with Cu_3_(HITP)_2_ and Co_3_(HITP)_3_. As the content of the higher conductivity metal (Co < Cu < Ni) increases, the electrical conductivity for the mixed metal MOF increased almost linearly with content. Chen et al. demonstrated a similar variation in electrical conductivity with Ni content between Ni_3_(HITP)_2_ and Co_3_(HITP)_2_ (Figure 20b). However, a poorer synthetic method led to inferior crystallinity in Co_3_(HITP)_2_, and the resulting bi‐metallic MOFs showed a preference for Ni over Co, evidenced by a higher Ni content than expected, given the proportions of precursor metals.^[^ ^34^ ^]^

Despite this, the effect on the *S* was not investigated in either study. As seen in Equation (1), S impacts the *zT* significantly more than *σ*, which means that this approach may still be viable in the design of enhanced TE MOFs, even if it does not yield more electrically conducting MOFs per se.

### 3.2 Linker Selection

Currently, a main advantage that MOFs offer for material design is the high modularity endowed to them by the organic linkers. The organic ligand can be carefully selected with different functionalities and tailored for different applications.^[^ ^57^ ^]^ To fully utilize this property of MOFs in order to design better‐performing TE materials, it is good to develop an understanding of the effect of varying the ligand structure on the resulting MOFs’ electronic characteristics, including structure, charge‐carrier pathways, thermal and electrical conductivities, and ultimately TE performance.

**Figure 21:**
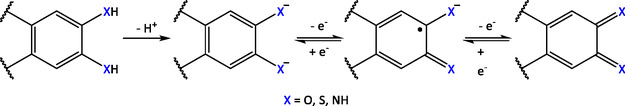
Deprotonation and subsequent redox reactions of chelating moiety present in HXTP and HXB linkers.

MOFs based on the HXTP and HXB linkers have been the most successful in imparting intrinsic electrical conductivity. These linkers feature aromatic, conjugated cores with chelating heteroatoms that are redox active. During the MOF synthesis, the linkers are deprotonated (by a base) and then form coordination bonds with the metal center, followed by subsequent oxidation of the ligand. Each chelating group: diol, diamine, and dithiol can undergo two consecutive oxidation steps/reactions (**Figure** **21**).^[^ ^57^ ^]^ As such, each linker is able to span seven charges (0 to −6). This results in MOFs that contain mixed‐valence linkers, enabling high electrical conductivities. EPR studies of a range of these MOFs with different metals have indicated the presence of organic radicals around the ligand, suggesting that the redox activity within these conductive MOFs is centered on the linkers rather than the metals.^[^ ^82^, ^85^ ^]^

This class of linkers already offers a wide variety of MOFs, with different heteroatoms and sizes/shapes of the conjugated organic core. The following section will discuss how varying the heteroatom and the size and shape of the conjugated core affect the conductivity of MOFs and their TE properties.

#### 3.2.1 Heteroatoms

The through‐bond transport pathway in MOFs emphasizes the importance of the metal–ligand interactions in enhancing electrical conductivity. Typically, MOFs are synthesized via carboxylic linkages, with hard oxygen heteroatoms on the linker, resulting in a large degree of ionic bonding. As such, these MOFs tend to be electric insulators. Changing the heteroatom to a softer and more electropositive atom, such as nitrogen or sulfur, will allow for better orbital matching between the metal and the linker, facilitating charge delocalization across a more covalent bond.

This effect of moving to a softer heteroatom can be demonstrated via comparison between Cu_3_(HHTP)_2_ and Cu_3_(HITP)_2_.^[^ ^64^, ^71^ ^]^ The electrical conductivity of the HITP MOF is 2 orders of magnitude greater than that reported for the HHTP‐based MOF. The HHTP MOF also has a significantly greater bandgap compared to HITP (2.68 and 0.291 eV, respectively). This highlights how the incorporation of the softer nitrogen heteroatom has improved metal‐linker interactions, providing greater dispersion in the band structure and resulting in a smaller band gap and higher electrical conductivity.^[^ ^64^, ^71^ ^]^ However, whilst this is most favorable for high electrical conductivities, it might have a negative impact on other TE parameters, such as a small *S* value resulting from a metallic band structure. Despite this, Cu_3_(HTTP)_2_ displayed an even lower electrical conductivity (2.4 × 10^−8^ S cm^−1^) than the Cu‐HHTP MOF.^[^ ^71^, ^86^ ^]^

Typically, for MOFs synthesized with both the nitrogen and sulfur analogs of the same linker, the sulfur analog has yielded higher electrical conductivity. This is most evident for Cu_3_(HTB)_2_, which has consistently yielded the highest electrical conductivity values, with a value two orders of magnitude higher than the nitrogen equivalent (HIB).^[^ ^41^, ^72^, ^79^ ^]^ This is consistent with Ni MOFs, where the HTB analog has a conductivity 20 times greater than the HIB MOF.^[^ ^72^, ^87^ ^]^

However, it should be noted that the as‐prepared Ni_3_(HTB)_2_ had a considerably lower conductivity (2.8 S cm^−1^) than oxidized Ni_3_(HTB)_2_. The as‐prepared Ni_3_(HTB)_2_ did not yield a charge‐neutral framework (as is the case for other 2D N‐based MOFs) and, as such, contained counterions. Complete oxidation to a charge‐neutral framework resulted in the loss of the counterions and a framework that exhibited higher electrical conductivities.^[^ ^87^ ^]^ The presence of counterions appears to be common in sulfur‐based HTTP‐MOFs, and electrical conductivities are significantly lower than those of the HITP analogs.^[^ ^81^, ^86^ ^]^ Furthermore, as demonstrated by Kambe et al. post‐synthetic complete oxidation of the MOF to yield a charge‐neutral framework could be implemented to improve the electrical conductivity of these MOFs.^[^ ^87^ ^]^

The requirement of oxidation to yield highly conductive frameworks was also demonstrated by Jiang et al.^[^ ^88^ ^]^ Despite NiTAA‐MOF being based on a HITP linker derivative as in the highly conductive Ni_3_(HITP)_2_ MOF, the addition of tetramethoxypropane yielded a framework that was insulating. This was attributed to the prevention of linker oxidation during MOF formation, as suggested by the lack of organic radicals in the EPR signal. However, upon doping with I_2_, the MOF demonstrated electrical conductivity more than 8 orders of magnitude higher, and the presence of organic radicals suggested the oxidation of the framework.^[^ ^88^ ^]^

The nature of the heteroatom of the linker plays a significant role in the metal‐linker interaction, thus having a significant influence on the through‐bond charge‐carrier pathway. Typically, implementing softer heteroatoms, for example, nitrogen and sulfur, has yielded MOFs with improved electrical conductivity attested to the improved metal‐linker interactions. Results have indicated that the sulfur heteroatom is most successful at optimizing the MOF electrical conductivity—however, there is limited computational analysis to support this. The sulfur‐based frameworks exhibit significant potential. Through further oxidation, these frameworks can achieve high *σ*, highlighting that the engineering of charge‐neutral frameworks represents another promising approach to enhancing MOF design. The impact of utilizing softer heteroatoms on the bandgap and *S* has also been understudied. Thus, whilst sulfur might be preferable for maximizing electrical conductivities, it might not consistently be optimum for yielding the optimized TE MOF.

#### 3.2.2 Heteroatoms and Effect on Crystallinity

Typically, the higher the degree of crystallinity of a conductive sample, the higher the electrical conductivity. Softer heteroatoms which form stronger bonds with the metals, that are less reversible, tend to result in MOFs with poorer crystallinity. Dou et al. designed the HHTT linker with the purpose of improving the crystallinity of the MOF by increasing the acidity of the OH group via the incorporation of electron‐withdrawing nitrogen atoms into the center of the organic aromatic moiety.^[^ ^89^ ^]^ This allowed the synthesis of a range of single‐crystal MOFs. Single‐crystal measurements for electrical conductivity reduce the effect of grain boundary resistances and give a better indication of the intrinsic conductivity of the MOF. Hence, Dou et al. were able to significantly improve the *σ* of the reported Ni–O and Cu–O based 2D MOFs; from polycrystalline 0.001 S cm^−1^ (Ni_3_(HHTP)_2_ and 3.8 × 10^−8^ S cm^−1^ (Cu_3_(HHTP)_2_) to single‐crystal 30 S cm^−1^ (Ni_3_(HHTT)_2_) and 80 S cm^−1^ (Cu_3_(HHTT)_2_).^[^ ^71^, ^90^ ^]^ This approach even afforded the second‐highest electrical conductivity for a Cu‐based 2D MOF despite the lack of softer heteroatoms. Despite this, these are still orders of magnitude lower than the highest *σ* achieved. Nevertheless, this study emphasizes the possibility of systematically designing ligands via functional groups, generating new MOFs tailored for TE applications.^[^ ^89^ ^]^

This highlights the significant role heteroatom selection can play in the design of MOFs. Softer heteroatoms promote strong metal‐linker bonds but can hinder crystallinity. On the contrary, more acidic heteroatoms can succeed in single‐crystal synthesis but are still limited by poorer metal‐linker interactions. Therefore, successful engineering of linkers to optimize metal‐linker bond strength for improved *σ*, while avoiding poor crystallinity, will aid in the development of intrinsically conductive MOFs.

#### 3.2.3 Ligand Size

The extended conjugation and through‐space charge‐transfer pathways both rely significantly on the degree of conjugation within the *ab* plane. The different triphenylene and benzene‐based linkers have conjugated aromatic cores of different sizes. Despite HXTP linkers having a larger aromatic core, MOFs based on HXB linkers have typically demonstrated larger electronic conductivities. This could be a result of the denser structures these smaller linkers afford. However, despite these higher conductivities, computational studies indicate that MOFs based on these materials may not be suitable for TE applications because they are predicted to have metallic character. Syrotyuk et al. computationally compared the Ni and Cu analogs of HITP and HIB linkers.^[^ ^59^ ^]^ The predicted band structures indicated that the HIB‐based MOFs would be metallic, whilst the HITP MOFs would be degenerate p‐type semiconductors. This would make HIB MOFs unfavorable for TE materials, resulting in low *S* values, such as for Cu_3_(HTB)_2_. On the contrary, Hinckley et al. recorded an exceptionally high *S* value for Cu_3_(HIB)_2_, suggesting non‐metallic character.^[^ ^72^ ^]^ This highlights the discrepancy that has often emerged in this field between computational and experimental results.

The HXB MOFs feature smaller pores compared with the HXTP analogs, ≈1 and ≈2 nm, respectively.^[^ ^32^, ^41^, ^81^ ^]^ This implies that HXB MOFs will have higher thermal conductivities than the HXTP MOFs due to less efficient phonon scattering by the pores. This can be seen from the large *κ* ascribed to Cu_3_(HTB)_2_ (1.99 W m^−1^ K^−1^). However, as previously mentioned, the exceptionally high *σ* (1580 S cm^−1^) reported for this MOF meant the major contributor toward the total thermal conductivity was the electronic component, determined via Wiedemann–Franz law. As such, the difference in the *κ*_l_ between the HXTP and HXB may be negligible compared to the achievable electrical conductivity.^[^ ^41^ ^]^

It appears that the linker size can have significant competing TE properties. Whilst the smaller, denser aromatic cores tend to yield higher electrical conductivities, these are also computationally predicted to result in the formation of MOFs with more metallic character (and thus smaller *S*), consequently yielding higher thermal conductivities than their larger counterparts. Such observations highlight the persistent nature of linker size on the engineering of TE properties while also demonstrating the need for further data generation.

#### 3.2.4 Interlayer Distance

The through‐bond pathway generally has the highest charge mobility in 2D MOFs. Despite this, in these 2D MOFs, the *π*–*π* stacking of the conjugated layers also allows for a through‐space conductivity pathway. This ensures conductivity pathways around defects and in grains that are not well‐aligned to the applied electric field, as well as current spreading from electrical contacts. However, while some computational studies have indicated that a smaller interlayer distance may increase the electrical conductivity, it can also have severe negative impacts on S. Li et al. reported from a computational study that the introduction of multiple layers into the stimulation resulted in a material with more metallic character compared to the monolayer, which had a band gap of 0.113 eV.^[^ ^91^ ^]^ This was attributed to the interlayer interaction involving orbital hybridization, which resulted in band splitting. Li et al. were able to further show the significance of tuning the interlayer distance and layer displacement on the electronic band structure for the Ni_3_(HTB)_2_ MOF. This showed that for bilayer MOFs, as the interlayer distance decreased, the bandgap was also reduced for both AA and AB stacking.^[^ ^91^ ^]^

**Figure 22:**
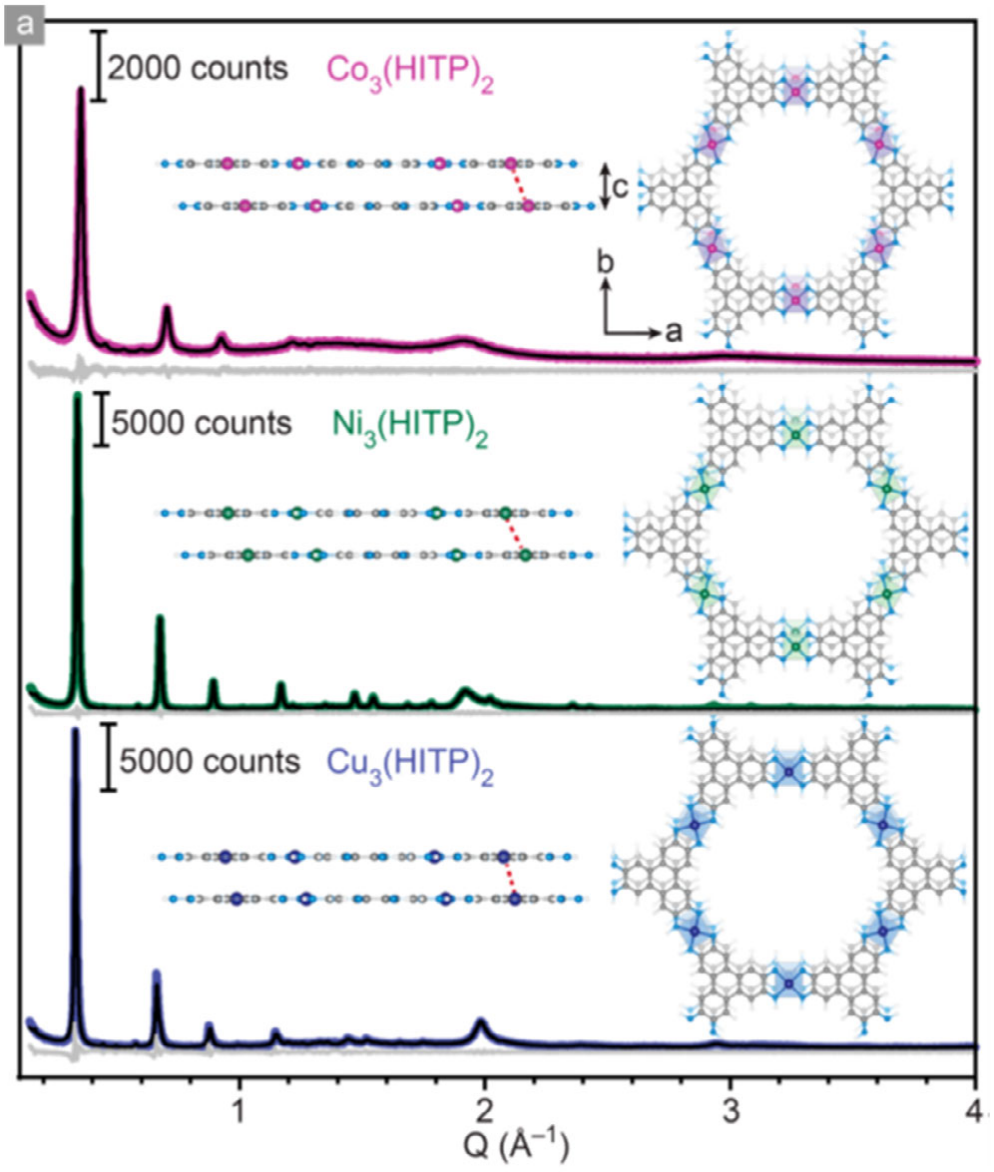
Synchrotron powder X‐ray diffraction patterns and corresponding Pawley refinements for Co_3_(HITP)_2_, Ni_3_(HITP)_2_, and Cu_3_(HITP)_2_, including insets of the simulated structures highlighting the shift in the interlayer distance and lateral interlayer displacements (red dashed lines) between layers with different metal cations.^[^ ^64^ ^]^ Reprinted (adapted) with permission.^[^ ^64^ ^]^ Copyright 2020, American Chemical Society.

The aforementioned bimetallic HITP‐based MOF study by Chen et al. also reflected the significance of the impact of metal ions on the interlayer distance and lateral interlayer displacement.^[^ ^64^ ^]^ This study attributed the difference in conductivity of the mono‐metallic MOFs (Co_3_(HITP)_2_, Ni_3_(HITP)_2_, and Cu_3_(HITP)_2_) to the difference in interlayer distance and lateral interlayer displacement (**Figure** **22**). The superior performance of Ni_2_(HITP)_3_, which yielded the highest conductivity (55.4 S cm^−1^), was attributed to the optimized lateral interlayer displacement (1.562 Å), which was the largest for the three MOFs (Cu_3_(HITP)_2_–0.869 Å and Co_3_(HITP)_2_–1.390 Å). While the slightly larger electrical conductivity of Cu_3_(HITP)_2_ (0.75 S cm^−1^) compared to the Co equivalent (0.024 S cm^−1^) was attributed to the significantly smaller interlayer distance (Cu_3_(HITP)_2_–3.16 Å, Co_3_(HITP)_2_–3.29 Å, and Ni_3_(HITP)_2_–3.23 Å). Incorporation of Ni at increasing percentages into Cu‐ and Co‐MOFs lead to an exponential increase in electrical conductivity that was attributed to a shift of the MOF structure toward an optimal interlayer distance and lateral interlayer displacement, seen for the Ni MOF.^[^ ^64^ ^]^

As such, while it is clear that a smaller interlayer distance is favorable for increasing electrical conductivity, the optimized lateral displacement of the MOF also plays a significant role in maximizing the MOF's electrical conductivity. It should, however, be noted that whilst interlayer distance and displacement certainly contribute to the refined electrical conductivity of the MOFs, this evaluation disregarded the impact of the electronic nature of the different metal ions—which would certainly influence the final electrical conductivity.^[^ ^64^ ^]^

**Figure 23:**
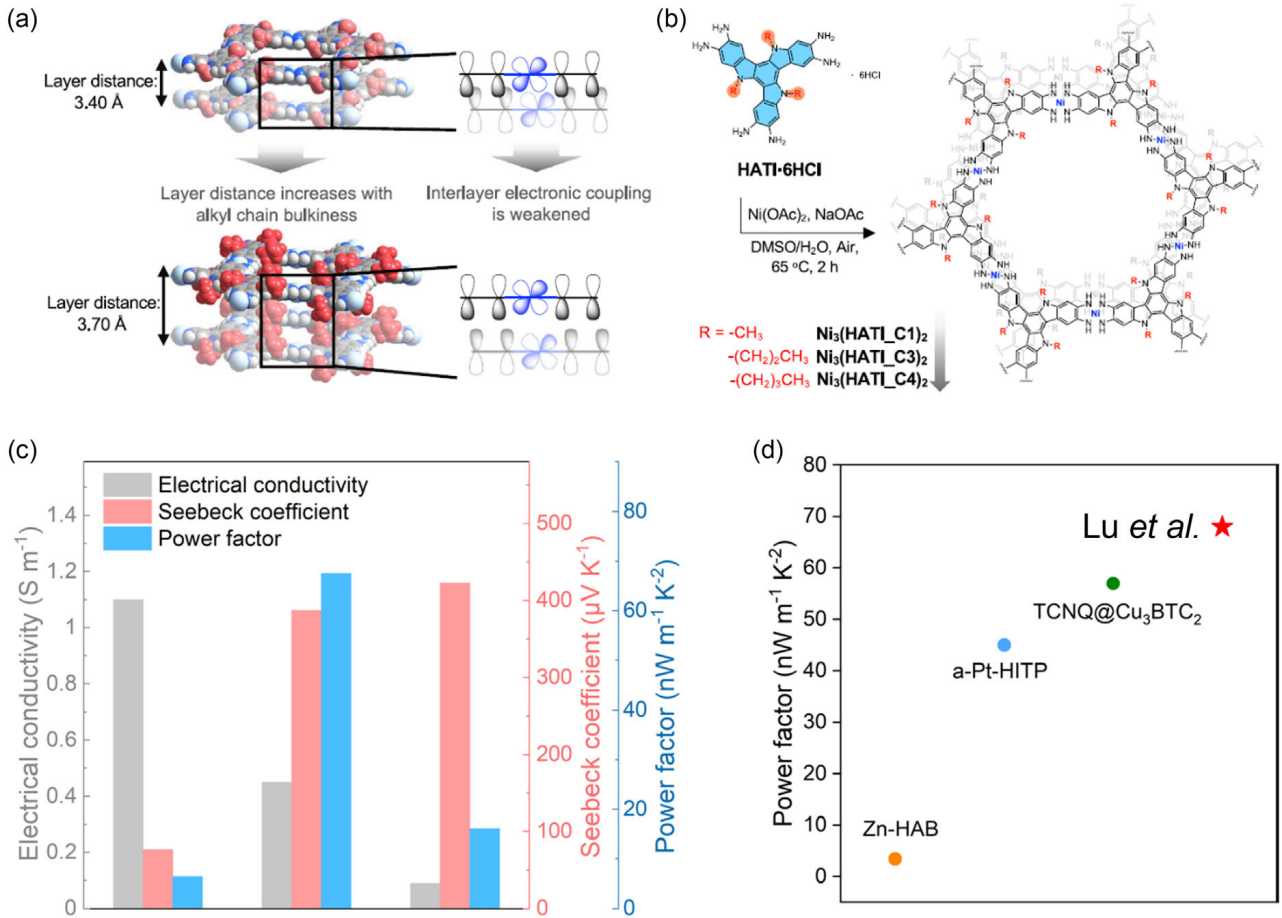
a) Space‐filling and atomic orbital diagrams indicating the structural change in the Ni_3_(HITP)_2_‐based MOF by varying the length of the alkyl chain on the modified HATI_Cx linker. b) Synthesis and chemical structure of modified Ni_3_(HATI_Cx)_2_ MOFs. c) Variation in the electrical conductivity, Seebeck coefficient, and power factor of the modified Ni_3_(HATI_Cx)_2_ by increasing alkyl chain length. d) Comparison of PF achieved compared to other studies on TE MOFs.^[^ ^92^ ^]^ Reprinted with permission.^[^ ^92^ ^]^ Copyright 2022, Nature Communications.

Lu et al. designed a series of 2D MOFs tailored toward tuning the interlayer distance.^[^ ^92^ ^]^ The HATI_CX linker (X number of carbons in the side chain) is based on the highly conductive HITP, with the incorporation of a pyrrole group into the aromatic backbone to enable additional functionality. Incorporating side chains of increasing length (**Figure** **23**) allowed for the tunable increase of the interlayer distance. This showed that the increasing interlayer distance (3.40, 3.68, and 3.70 Å) significantly decreased the MOF electrical conductivity due to the weakened through‐space interaction (Figure 23). However, this was coupled with an increase in *S* and notably: there was only a partial increase in *S* between 3.68 and 3.70 Å, compared to a more pronounced decrease in electrical conductivity. As such, the calculated PF values for all three MOFs indicated an optimum interlayer distance for achieving an efficient TE MOF, where the *S* is improved but without significantly impeding the electrical conductivity.^[^ ^92^ ^]^ With this, Lu et al. were able to achieve the second‐highest PF reported thus far for MOFs at 0.0068 μW m^−1^ K^−2^.^[^ ^92^ ^]^

**Figure 24:**
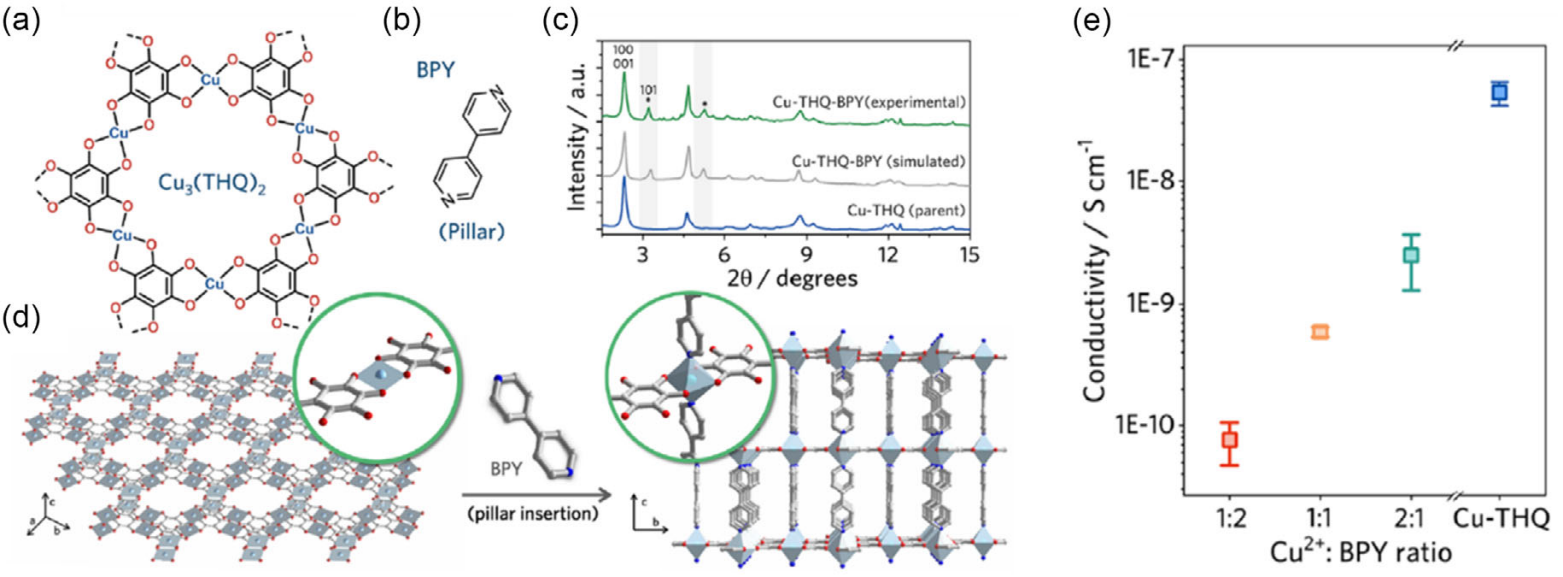
a) Structure of Cu_3_(THQ)_2_. b) Structure of pillar linker. c) Powder X‐ray diffraction patterns of Cu_3_(THQ)_2_ before and after pillar linker insertion compared to simulated. d) Structural change of Cu_3_(THQ)_2_ MOF upon pillar linker insertion. e) Changes in the electrical conductivity are caused by varying ratios of pillar linkers. Electrical conductivity is seen to decrease with the increased ratio of pillar linkers.^[^ ^95^ ^]^ Reprinted (adapted) with permission.^[^ ^95^ ^]^ Copyright 2020, American Chemical Society.

The expansion of layered materials via the insertion of guests between layers has been adapted for various materials, including graphene and Hofmann‐type clathrates.^[^ ^93^, ^94^ ^]^ This pillar linker insertion had been previously reported computationally by Foster et al. to increase interlayer distance in Ni_3_(HITP)_2_ to yield a material with semiconducting rather than metallic properties.^[^ ^63^ ^]^ This was then investigated experimentally with the Cu_3_(THQ)_2_ MOF. The Cu node in this MOF exhibits a preference for either square planar or octahedral geometries. As a result, by selecting a nitrogen donor ligand (BPY), the pillar ligand could be inserted into the 2D MOF via coordination of the nitrogen group to the square planar Cu to yield a 3D MOF with octahedral geometry at the metal center. This afforded an increase in the interlayer distance, which decreased the *π*–*π* stacking interaction and decreased the electrical conductivity, **Figure** **24**.^[^ ^95^ ^]^ There was a degree of tunability on the effect of pillar insertion via control of the Cu:BPY ratio. As the proportion of BPY increased, the electrical conductivity was reported to decrease significantly. Despite this, no significant changes in the MOF's bandgap upon pillar linker insertion, determined via UV‐vis‐NIR spectroscopy and Tauc plots, were reported. However, an increase in the thermal activation barrier (*E*_a_ = 0.17–0.29 eV) was determined. Previous computational studies by Foster et al. would indicate that an increase in the bandgap is favorable for high *S*.^[^ ^63^ ^]^

When striving to obtain MOFs with maximized electrical conductivity, the smallest interlayer distance is preferred for enhancing the through‐space charge pathways. However, this has often resulted in metallic frameworks, and thus poor *S* values. As such, techniques for expanding the interlayer distance, via linker design or addition of spacer linkers, appear effective in reducing the electrical conductivity.

### 3.3 Materials Engineering

#### 3.3.1 Compositing

**Figure 25:**
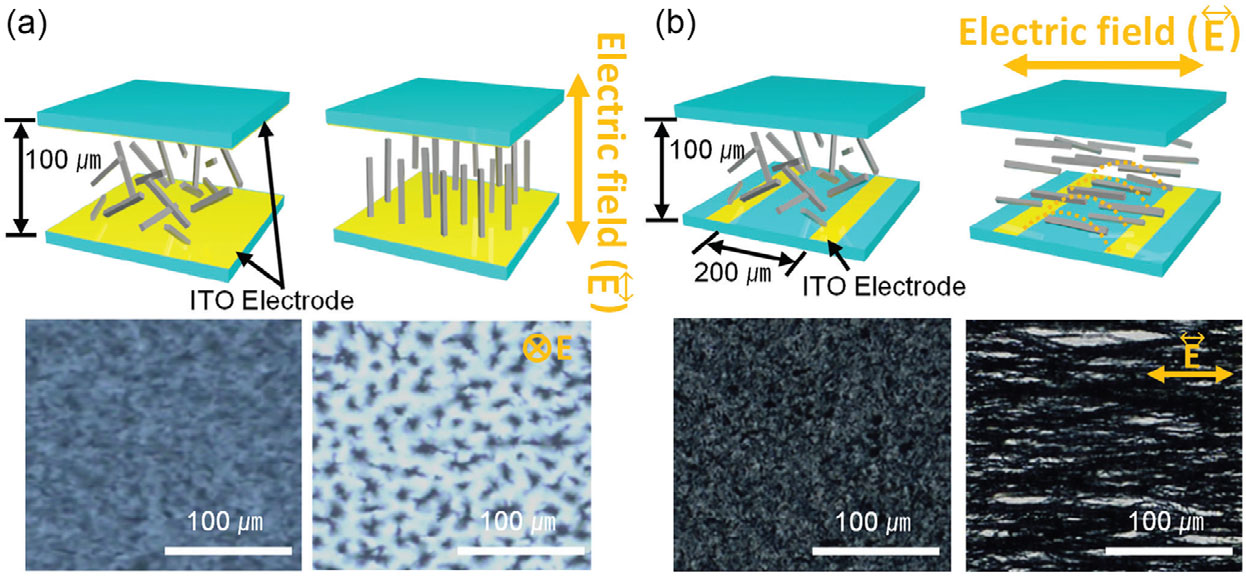
a) Perpendicular alignment of MOF crystal rods in a magnetic field and the resulting optical images demonstrate alignments; b) Parallel alignment of MOF crystals in a magnetic field and the resulting optical images demonstrate alignment.^[^ ^96^ ^]^ Reprinted (adapted) with permission.^[^ ^96^ ^]^ Copyright 2024, John Wiley and Sons.

Typically, intrinsically conductive MOFs feature anisotropic crystal structures and, as such, result in anisotropic charge transport pathways. This could severely impact the measured bulk electrical conductivity of pelletized samples if there is a significant lack of alignment. Hong et al. recently demonstrated the ability to post‐synthetically tune the alignment of intrinsically conductive Cu_3_(HHTP)_2_ samples via dielectrophoresis. Cu_3_(HHTP)_2_ powders were suspended in the insulating PEGDA oligomer matrix. An electric field was applied to the matrix, resulting in the alignment of the Cu_3_(HHTP)_2_ within the matrix (**Figure** **25**), as seen from the optical microscope images. The electric field allowed for the alignment of the MOF, however, also generated an internal magnetic field of the aligned crystals resulting in self‐aggregation. Hence, photopolymerization of the PEGDA polymer allows for the fixation of MOF crystals, maintaining alignment without the presence of the magnetic field.^[^ ^96^ ^]^

**Figure 26:**
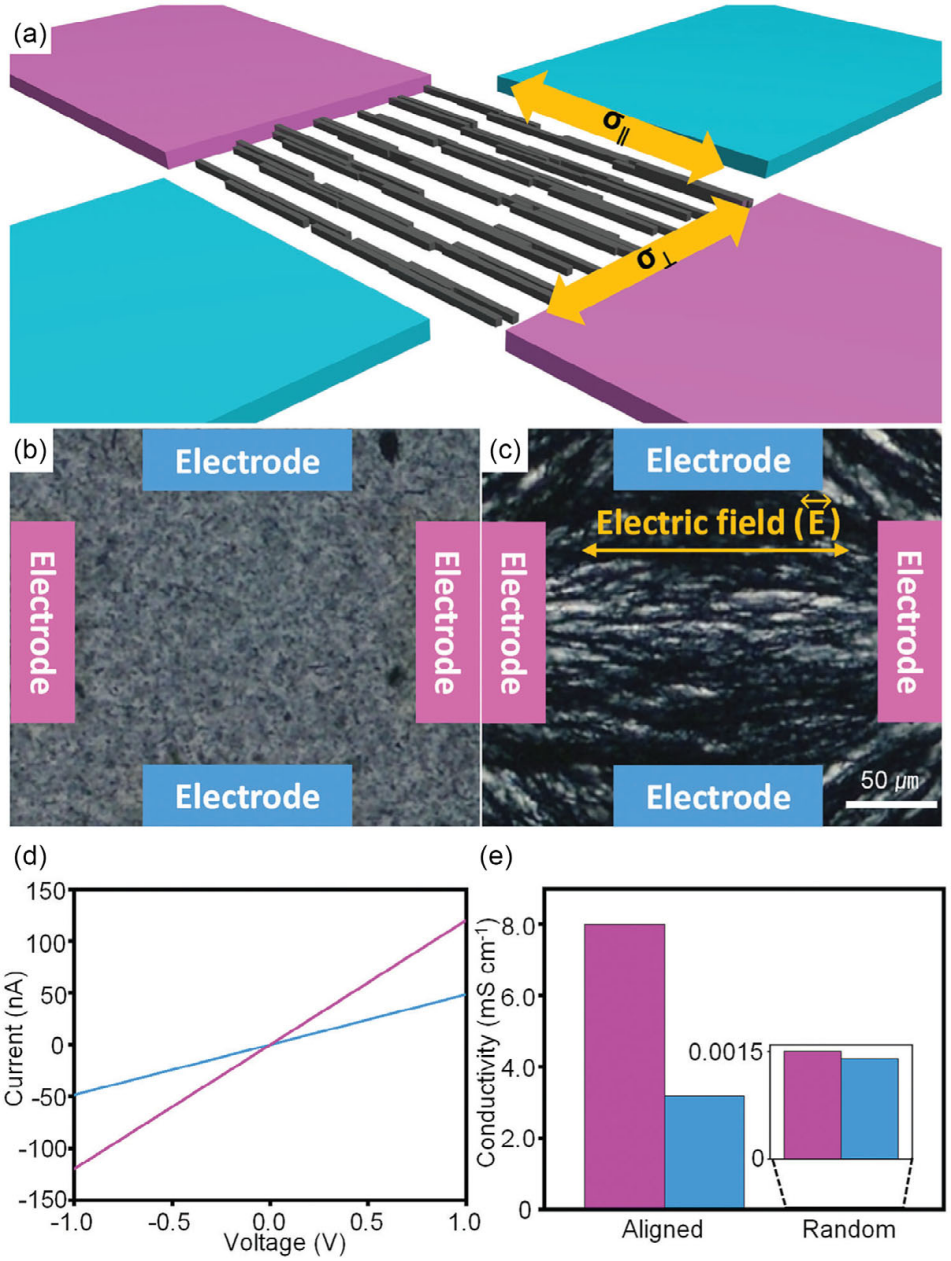
a) Illustration of the configuration used to measure MOF‐polymer composite film's parallel and perpendicular electrical conductivity. Optical images illustrating the setup for the b) unaligned film and c) parallel‐aligned film. d) Ohmic *I*–*V* curves measured within ±1 V of an open circuit; pink and blue lines correspond to the measurements performed across parallel and perpendicular directions to the alignment direction, respectively. e) Comparison of the parallel and perpendicular electrical conductivities measured for the aligned and unaligned films.^[^ ^96^ ^]^ Reprinted (adapted) with permission.^[^ ^96^ ^]^ Copyright 2024, John Wiley and Sons.

The electrical conductivity was measured both parallel (*σ*_∥_) and perpendicular (*σ*_⊥_) to the direction of the alignment (**Figure** **26**). Alignment resulted in an improvement in both the parallel and perpendicular conductivity of more than three orders of magnitude (8.0 × 10^−3^ and 3.2 × 10^−3^ S cm^−1^, respectively), compared to the unaligned composite (1.5 × 10^−6^ and 1.4 × 10^−6^ S cm^−1^, respectively). The significant improvement in the parallel electrical conductivity can be attributed to both the alignment of the crystals and the self‐aggregation, allowing for improved charge‐carrier pathways through the insulating matrix. In contrast, the improvement in the perpendicular direction is limited to only the self‐aggregated pathways. However, increasing the relative concentration of the MOF within the matrix hindered the alignment of the crystals. This was due to neighboring crystals hindering the rotation to allow complete alignment, and as such, a decrease in the measured electrical conductivity was observed above 1 wt% of MOF. Despite this low weight percent, the electrical conductivity for the aligned MOF‐polymer composite was within the same order of magnitude as electrical conductivity measurements on bulk pelletized Cu_3_(HHTP)_2_ samples. This highlights the successful post‐synthetic modification of intrinsically conductive MOFs for improved electrical conductivity—without doping.^[^ ^96^ ^]^

#### 3.3.2 Morphology Engineering

Typically, in semiconductors for TE, low thermal conductivities have been achieved via nano‐structuring, that is, through the introduction of grain boundaries and defects. However, these methods also negatively impact electrical conductivity. The introduction of intrinsic porosity to an intrinsically conductive material could help optimize the *σ*/*κ* ratio by helping to decouple their interdependence. This has been utilized in other porous electrically conductive materials to yield TE materials.^[^ ^97^, ^98^, ^99^, ^100^ ^]^ However a distinct disadvantage there is often the lack of control of porosity and nanostructuring, which can interfere with the material band structure thus hindering electrical conductivity. In contrast, the high crystallinity of MOFs results in structures which are well‐defined and predictable including their high intrinsic porosity. As such, MOFs offer the unique potential to manipulate electronic and thermal conductivity independently with defined control over their structure and porosity.

**Figure 27:**
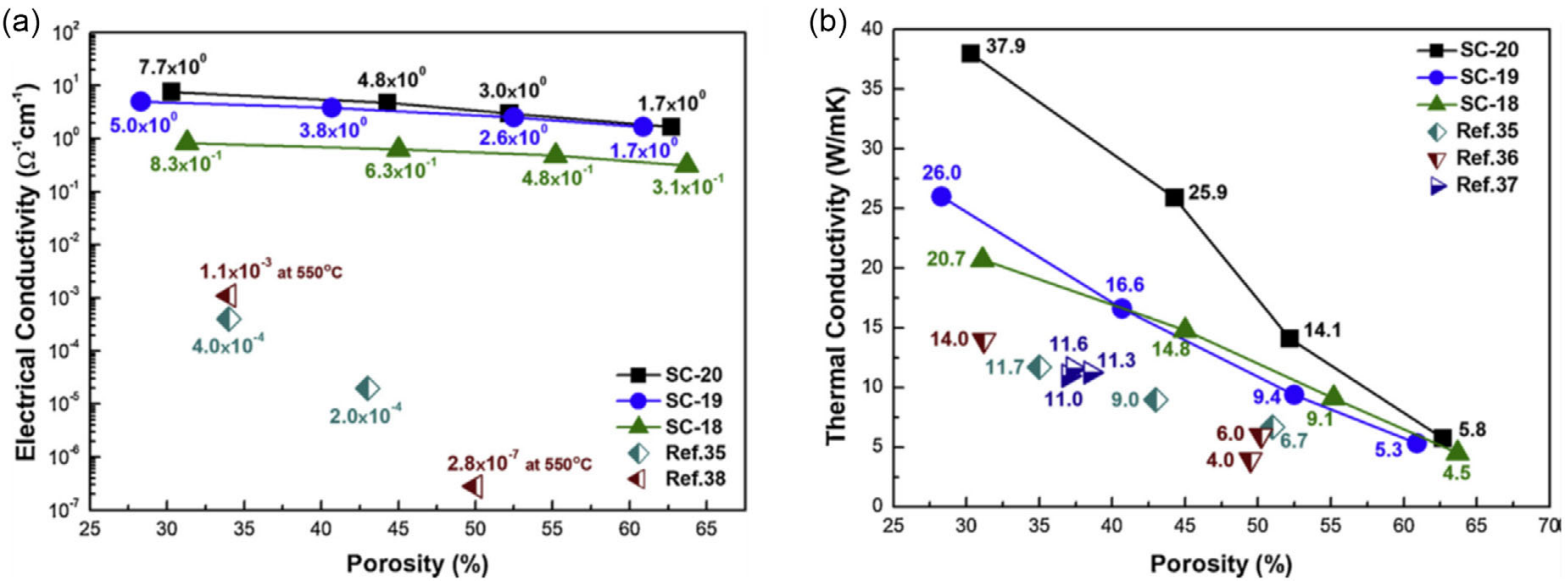
The effect of increasing porosity on a) the electrical conductivity and b) the thermal conductivity for a series of SiC ceramics with increasing grain size (SC‐20 > SC‐19 > SC‐18).^[^ ^101^ ^]^ Reprinted (adapted) with permission.^[^ ^101^ ^]^ Copyright 2020, John Wiley and Sons.

For example, a study on a series of silicon carbide ceramics demonstrated that grain size and porosity had different impacts on the electrical and thermal conductivities.^[^ ^101^ ^]^ Increasing the porosity and decreasing grain sizes both resulted in a general decrease in the thermal and electrical conductivities (**Figure** **27**). However, it was found that grain morphology, over porosity, had a more significant impact on the electrical conductivity of silicon carbide ceramics, whereas porosity influenced thermal conductivity more. This allowed for a decoupling of the electrical and thermal conductivities.

This demonstrates how manipulation of the materials’ porosity versus grain size could enable the engineering of a phonon‐glass electron‐crystal material by tailoring for a material with both high crystallinity and high porosity—for which MOFs are an ideal platform. However, this would also require a greater understanding of the mean free pathways for both phonons and electrons in a given material and how these would be impacted by MOF porosity and grain morphology.

### 3.4 Machine Learning for Thermoelectric MOF Screening

The implementation of machine learning (ML) models has revolutionized material design by enabling high‐throughput screening for new materials. Whilst there are currently growing databases of MOFs (e.g., CoREMOF and QMOF), these are still of limited size in comparison to other material types.^[^ ^102^, ^103^ ^]^ This is largely owing to the difficulties in the accurate collection of data, due to the complex structural and chemical diversity of MOFs. Obtaining accurate data to form large, extensive databases is computationally demanding via molecular simulations and also requires accurate chemical and electronic structures from experimental studies. Properties requiring density functional theory (DFT) calculations, like band gaps—crucial for electrically conductive and TE MOFs—demand substantial time and computational resources, making it challenging to obtain large, accurate datasets efficiently.^[^ ^104^ ^]^

To the best of the authors’ knowledge, there is currently no literature specifically on machine learning techniques for thermoelectric MOFs. However, extensive reviews cover the development of ML models for MOFs, including properties such as thermal conductivity, as well as thermoelectric materials, which are beyond the scope of this review.^[^ ^104^, ^105^, ^106^ ^]^ The lack of ML models for electrically conductive and thermoelectric MOFs is partly due to the scarcity of experimental data required to train these models, particularly single‐crystal measurements for accurate structural and intrinsic electronic properties, coupled with the significant computational demands.

**Figure 28:**
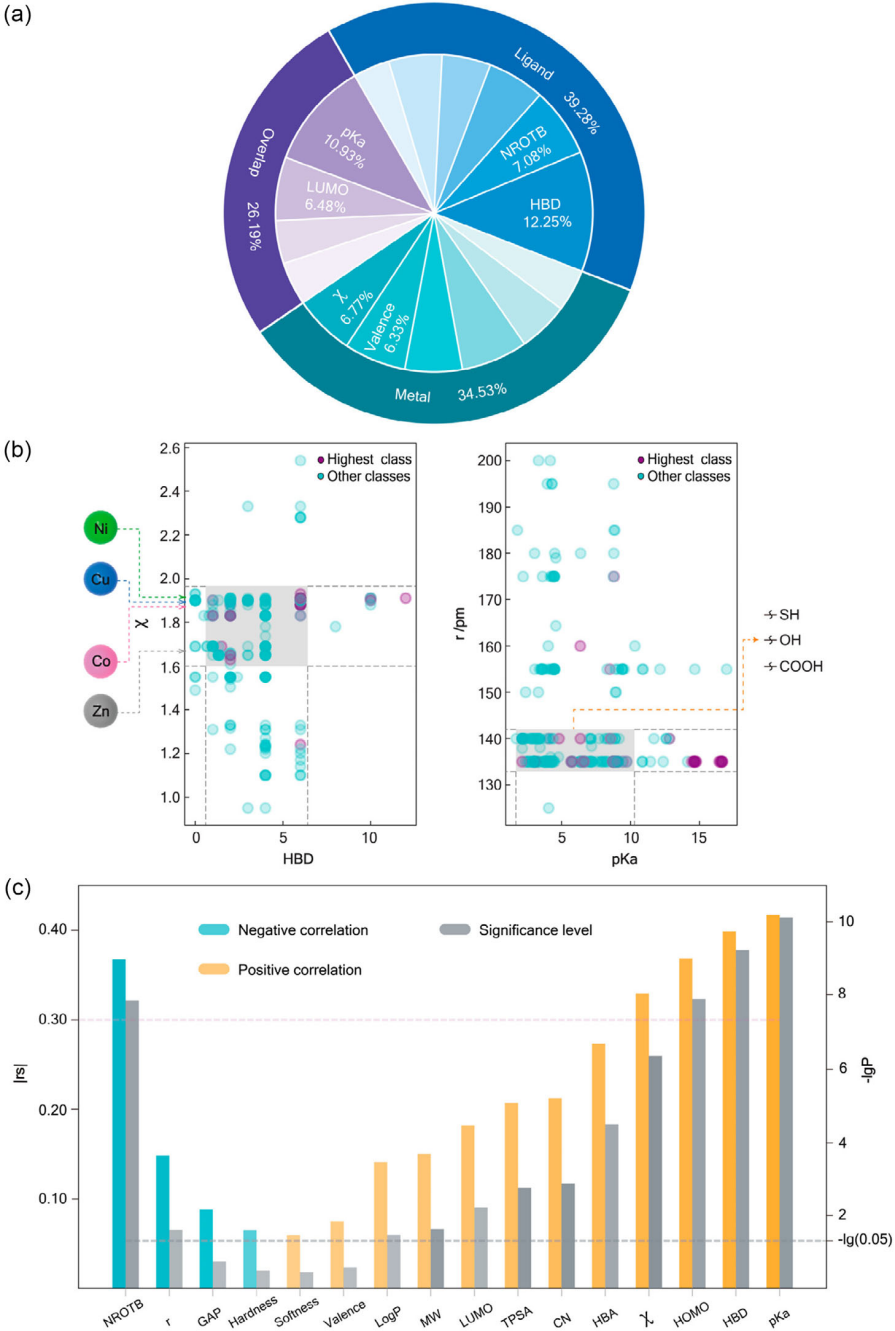
ML model interpretability. a) Importance distribution and ratios (calculated based on ensemble model) for each of the features within the three MOF descriptors (ligand, metal, and overlap) with the two most significant features in each descriptor annotated. b) For the four significant features, the distribution of the CM database is under six categories. The most conductive class of MOFs is represented by purple dots, and the remaining classes are represented by cyan dots. The gray region indicates the aggregations of highly conductive MOFs. c) |*rs*| and confidence level for different features. Negatively correlated features are represented by cyan bars, and positively features are represented by orange bars.^[^ ^107^ ^]^ Reprinted (adapted) with permission.^[^ ^107^ ^]^ Copyright 2024, American Chemical Society.

Despite this, Lin et al. recently developed the beginnings of an accurate ML model for conductive MOFs.^[^ ^107^ ^]^ ML models were trained from a database of 224 MOFs, experimentally tested for electrical conductivity, and utilized three descriptors; metal descriptor—electronic property, redox activity, and physical properties of the metal ions, ligand descriptor—physical and chemical information and degree of conjugation of the ligand, and the overlap descriptor—degree of metal‐ligand orbital overlap as calculated by density functional calculation (**Figure** **28**). The best performing of these models achieved accuracy of 90.48%, and when applied to the aforementioned QMOF database yielded the CuTTPD (5,5’‐(1,3,6,8‐tetraoxo‐1,3,6,8‐tetrahydrobenzo[*lmn*][3,8]‐phenanthroline‐2,7‐diyl)diisophthalic acid) MOF with predicted electrical conductivity (10^−3.30^ S cm^−1^). Experimental verification for CuTTPD yielded electrical conductivity within two orders of magnitude. This discrepancy could be accounted for by experimental variation (grain boundaries) or could reflect the need to optimize the models further.^[^ ^107^ ^]^

Nevertheless, whilst ML models might not yet be advanced enough to yield high‐throughput screening of TE MOFs of sufficiently high electrical conductivity, they could yield insight for the design of new conductive frameworks. Lin et al. assigned an importance score to each feature in the descriptors by assessing its contribution to the predicted outcome (Figure 28). Thereby, they identified four parameters with strong positive correlation: *pK*_a_ (acid dissociation constant), HBD (number of hydrogen bond donors), HOMO (highest molecular orbital) and *χ* (electronegativity of metal), and one parameter with strong negative correlation: NROTB (number of rotatable bonds). Several of these parameters have been identified and rationalized in the literature, as previously discussed in this review. Low NROTB corresponds to more rigid ligands with higher conjugation, resulting in better *π*–*π* stacking and conjugation pathways. Meanwhile, higher ligand HOMO and larger metal ion *χ* facilitate improved metal‐ligand orbital overlap. However, Lin et al.'s ML models identify HBD and *pK*_a_ as having the most significant influence on MOF conductivity. Lin et al. correlated these factors to be representative of redox activity of the coordination sites of the ligands studied. It was noted however, that consideration of the ligand redox activity should also account for the redox properties of ligand core, which might impact that relevant to the metal‐ligand energy overlap that was identified by these ML models.^[^ ^107^ ^]^

Nevertheless, this study represents a promising starting ground for the development of accurate ML models for both high electrical conductivity MOFs and eventually TE MOFs. This could then lead to the rapid acceleration of the TE MOF field by rapid material screening. However, for this to be achieved the current database of electrically conductive MOFs and TE MOFs much first be significantly expanded.

## 4 Conclusion

Despite the large structural diversity of MOFs, the range of intrinsically conductive MOFs is still limited to only a few metals (Co, Cu, and Ni) and linkers (HXB and HXTP, where X = O, S, or N), with only a few exceptions. Of these, an even smaller assortment has been assessed as TE materials. Whilst some exceptionally high electrical conductivities have been achieved (1580 S cm^−1^ for Cu_3_(HTB)_2_), most intrinsically conductive MOFs, for TE materials, are still limited by poor *σ* and low *S* values (particularly due to their computationally predicted metallic band structure). This has resulted in low PF and, for materials were *κ* has been determined, low *zT*, thus limiting the incorporation of MOFs in TE devices.

Furthermore, achieving good quality crystalline samples remains a challenge. The lack of crystalline samples has hindered measurements of *κ* such that only a few MOFs can report *zT*; although, there is advancement in measurement techniques for polycrystalline samples. This has also hindered the understanding of intrinsic transport mechanisms occurring in MOFs, owing to the lack of single‐crystal electrical conductivity measurements. Hence, it is critical in the development of new TE MOFs, to also strive for improving synthetic protocols to achieve highly crystalline samples. Not only should crystalline samples have improved *σ*, by limiting grain boundaries, there should not be a significant increase in *κ*_l_ owing the high porosity of crystalline MOFs. Therefore, measurements of polycrystalline samples have also been hindered by anisotropic *σ* in 2D layered MOFs. Alignment of conductive crystallites via magnetic field could also be utilized to boost performance.

Another major challenge faced in the design of TE MOFs is the current discrepancy between experimental data, computational data, and even between different computational methods. This limits our understanding of the structure–function relationship associated with MOFs, which is crucial for developing band engineering strategies for optimized TE properties. High throughput systematic computational screening, similar to that already done for thermal conductivity, of intrinsically conductive MOF structures, for optimized TE behavior, could be paramount to the development of TE MOFs. There is ongoing work to design models to improve computational accuracy, whilst minimizing computational load. This, coupled with ensuring accurate input material crystal structures, including defects, and expected disorder, will help the field to achieve more accurate computational results, thus advancing our understanding.

Design strategies for maximizing *σ* for intrinsically conductive MOFs have been subject to significant research; and although these are also important for the development of TE MOFs, more nuance is required. So far, experimentally, the larger aromatic core of HXTP yields frameworks of lower *σ* in comparison to HXB, however, these smaller linkers are predicted to be more metallic. Likewise, the larger pore of HXTP is more favorable for achieving low *κ*. The pore size also plays a role in imparting ultra‐low *κ*_l_ in a framework, but the intrinsically high porosity of MOFs is considered sufficient. As such, whilst HXB shows most potential for *σ*, appropriate development of a HXTP‐based MOF could still be significant for TE.

The careful selection of both linker and metal is paramount for achieving TE MOFs with high *zT*. Whilst there are a range of studies looking at various metal cations and linkers (heteroatoms and linker size), understanding of the optimum metal‐linker pairing is limited. Linkers with softer, more electropositive heteroatoms are favorable for better energy matching with the metal cations, allowing for more efficient charge transfer. However, this improved metal‐linker interaction has also typically been associated with reduced bandgaps and thus smaller *S*. Despite sulfur as the heteroatom seeming to be the most successful, yielding the highest *σ* in MOFs (e.g., Cu_3_(HTB)_2_), many of the structures synthesized with a sulfur heteroatom have not been fully optimized. This indicates the significant impact in yielding charge‐neutral frameworks for maximizing *σ*. From the MOFs reported thus far, the Cu–S pairing appears to have the optimum metal‐linker interaction for high *σ*, whilst Ni is best for nitrogen‐based MOFs. However, there has been a lack of systematic experimental studies surveying the range of metal‐linker interactions to fully attest the optimum linker pairing. For TE MOFs, this would also include full characterization of the impact of different metal‐linker pairings on the band structure and thus *S*, whilst also aiming for high *σ*. This coupled with the high‐throughput computational screening would significantly aid the design of efficient TE MOFs by further developing our understanding of the structure–function relationship.

Development of the structure–function relationship will also allow for band engineering via chemical and structural design. So far underutilized, the incorporation of 2^nd^ or 3^rd^ row transition metals appears to be a promising strategy for increasing both *σ* and *S*, with increased band dispersion, owing to the improved metal‐linker orbital interactions. The use of larger, heavier metal ions was also suggested as a strategy for decreasing *κ*. Thus, if samples of improved crystallinity can be achieved, noble metal‐based intrinsically conductive MOFs are of promise for TE materials. Whilst other strategies to yield higher *σ* frameworks have not been as successful, they still could offer potential for TE MOFs. Bimetallic MOFs did not observe a boost in *σ*, however *S* for these frameworks was not reported, and as *S* has a more significant impact on PF, even if *S* also increases linearly, could still observe an enhanced performance. Likewise, the use of mixed linkers (e.g., HTTP, HITP, or HHTP) could also be investigated. It is clear that the *π*–*π* interaction between stacking layers of 2D intrinsically conductive MOFs can offer more precise tuning of the materials band structure, by manipulation of the interlayer distance. Design tailored for optimizing a 2D structure for optimized PF is therefore also promising.

In summary, the analysis of intrinsically conductive MOFs for TE materials has revealed both significant advancements and ongoing challenges. While the structural diversity of MOFs offers a promising platform for enhancing TE performance, the limited range of metals and linkers that exhibit intrinsic conductivity underscores the need for further research. Future efforts should focus on optimizing metal‐linker interactions and improving crystallinity to unlock the full potential of these materials. By addressing these challenges, the development of highly efficient, intrinsically conductive MOFs could pave the way for innovative applications in TE devices.

## Conflict of Interest

The authors declare no conflict of interest.

## Author Contributions

**Molly McVea**: data curation (lead); formal analysis (lead); writing—original draft (lead). **Christian B. Nielsen**: writing—review & editing (supporting). **Oliver Fenwick**: writing—review & editing (equal). **Petra** **Ágota Szilágyi**: writing—review & editing (equal).
